# Implementation strategies to increase the uptake and impact of molecular WHO-recommended rapid diagnostic tests: evidence from a mixed-methods systematic review

**DOI:** 10.1136/bmjgh-2024-018700

**Published:** 2025-09-17

**Authors:** Ruvandhi R Nathavitharana, Abarna Pearl, Matthew O’Bryan, Matthew Edwards, Helene-Mari van der Westhuizen, Bruna Voldman, Advaith Subramanian, Naveed Delrooz, Omolayo Anjorin, Amanda Biewer, Carl-Michael Nathanson, Nora Engel, Nazir Ismail, Andrew McDowell, Karen Steingart

**Affiliations:** 1 Harvard Medical School, Boston, Massachusetts, USA; 2 Beth Israel Deaconess Medical Center, Boston, Massachusetts, USA; 3 Harvard School of Public Health, Boston, Massachusetts, USA; 4 Case Western Reserve University School of Medicine, Cleveland, Ohio, USA; 5 Primary Care Health Sciences, Oxford University, Oxford, UK; 6 Technion’s American Medical School, Haifa, Israel; 7 Tulane University, New Orleans, Louisiana, USA; 8 George Mason University, Fairfax, Virginia, USA; 9 World Health Organization, Geneva, Switzerland; 10 Maastricht University, Maastricht, The Netherlands; 11 University of the Witwatersrand Johannesburg, Johannesburg, South Africa; 12 Department of Clinical Sciences, Liverpool School of Tropical Medicine, Liverpool, UK

**Keywords:** Tuberculosis, Diagnostics and tools

## Abstract

**Introduction:**

Fewer than 50% of people with tuberculosis receive a molecular WHO-recommended rapid diagnostic test (mWRD). We performed a mixed-methods systematic review to categorise barriers and enablers that affect mWRD use and impact and evaluate mWRD implementation strategies. Parts of this review informed the WHO standard: Universal Access to Tuberculosis Diagnostics.

**Methods:**

We searched multiple databases without language restrictions until 29 July 2022. We included studies that used qualitative, quantitative or mixed methods study designs. Four reviewers independently screened studies and extracted data. We categorised studies as thick or thin depending on whether authors analysed findings beyond a descriptive list of barriers or enablers and demonstrated insights into participants’ perspectives. We appraised study quality by adapting the Standards for Reporting Implementation Studies statement. We synthesised data using a thematic approach and used GRADE-CERQual to assess confidence in the findings.

**Results:**

We identified 54 high-thickness studies from 18 countries, including public and private healthcare settings. Implementation strategies included engaging patients, training and supporting clinicians, building infrastructure and interactive assistance. Examples included remote outreach programmes, community testing, longitudinal clinician engagement, auxiliary workers, multicomponent strategies, performance feedback, improving health information management to strengthen care linkage and diagnostic network improvement. We had high or moderate confidence in our findings.

**Conclusion:**

Innovative and contextually relevant implementation strategies are needed for tuberculosis programmes to realise the benefits of improved accuracy and diagnostic expediency that mWRDs offer. Multicomponent strategies that centre equity and longitudinal health worker training across the diagnostic cascade must be prioritised.

**WHAT IS ALREADY KNOWN ON THIS TOPIC**

- A prior systematic review identified 11 studies with barriers and enablers to Xpert implementation and a prior qualitative evidence synthesis identified 32 studies that focused on user perspectives and experiences related to molecular WHO-recommended rapid diagnostic test (mWRD) implementation, but neither focused on implementation strategies and included fewer countries and testing contexts.

**WHAT THIS STUDY ADDS**

- We identified the importance of innovative, contextually relevant multicomponent strategies that address multiple barriers to different diagnostic cascade steps. Examples included remote and community outreach to expand the reach of testing, building infrastructure and systems to improve test access, training and supporting clinicians or other health workers to optimise test use, use of interactive assistance, mobile technologies and performance feedback to ensure people with tuberculosis receive results and are linked to treatment.

**HOW THIS STUDY MIGHT AFFECT RESEARCH, PRACTICE OR POLICY**

- The WHO Standard: Universal Access to Rapid Tuberculosis Diagnostics is a set of benchmarks that countries can use to improve access to tuberculosis diagnostics. The findings of this systematic review directly informed the Standard by mapping enablers, approaches and solutions which can be used at the facility and programme level to increase the use of mWRDs and close the tuberculosis diagnostic gap.

## Background

 Tuberculosis remains one of the leading global causes of death from a single infectious agent. In 2022, an estimated 10.6 million people became sick with tuberculosis and 1.3 million people died due to tuberculosis.^1^ Analyses of the tuberculosis (TB) cascade of care demonstrate losses to follow-up at each step of a patient’s care journey, but diagnosis consistently represents the largest point of systemic failure.^2^,^4^ The impacts of the COVID-19 pandemic widened this diagnostic gap. The same year, 3.1 million people were estimated to develop TB disease but were not notified to national health systems^1^; most of the missing people were presumed to be undiagnosed. Missed and delayed diagnoses increase individual disease severity and transmission.

The WHO first recommended molecular WHO-recommended rapid diagnostic tests (mWRDs) to diagnose TB and detect drug resistance to rifampicin in 2011.^5 6^ Between 2011 and the present, WHO guidelines have evolved from recommending that mWRDs be prioritised for people living with HIV and people at high risk of developing drug-resistant TB to recommending that mWRDs be undertaken as the initial test for all persons being evaluated for TB.^7^ However, the implementation, use and scale-up of mWRD testing remains limited. In 2022, despite the WHO recommendations and increased availability of these tests, only 47% of people newly diagnosed with TB were tested with an mWRD as their initial diagnostic test.^1^

Patient pathway analyses demonstrate that people with TB encounter circuitous care pathways, highlighting many missed opportunities for diagnosis and engagement in care.^8^ There is increasing recognition that diagnostic tests only improve patient outcomes if they are available to and valued by health workers, who then implement the test, interpret results correctly and engage people in care.^9 10^ Implementation barriers affecting mWRD use and scale-up vary according to clinical and country setting and have been described in a previous qualitative evidence synthesis by Engel *et al*.^10^ These barriers include insufficient test availability and infrastructure, inadequate healthcare provider training and capacity, as well as limited national and local-level agency in policy decision-making.^10^ Conversely, implementation enablers include expanding diagnostic algorithms and optimising sample transport.^11^ Implementation strategies to improve the use of mWRDs have not been systematically evaluated.

Combining quantitative and qualitative evidence can help decision makers understand intervention complexity and contextual variability in implementation approaches and stakeholder perspectives.^12 13^ To this end, we performed a mixed-methods systematic review to synthesise evidence on implementation strategies to increase the use or impact of mWRDs to diagnose TB and detect drug-resistant TB. This systematic review was commissioned by the WHO to inform the WHO standard: Universal Access to Rapid Tuberculosis Diagnostics.^14^ We aimed to examine barriers and enablers affecting the use or impact of mWRDs, evaluate how implementation strategies address barriers and how implementation strategies affect mWRD implementation, service and user outcomes (online appendix).

## Methods

### Types of studies

We registered the review protocol on 21 September 2022 on the International Prospective Register of Systematic Reviews (ref CRD 356771). We included research studies that used qualitative, quantitative or mixed methods study designs. We included studies with relevant data on mWRD implementation for diagnosing TB and drug resistance from the perspective of a range of stakeholders, including patients, healthcare providers, laboratory technicians, implementers and policymakers. We included studies relevant to mWRD implementation conducted in low- and middle-income or high-income countries. We included operational reports but excluded conference abstracts and narrative reviews.

### Search strategy

We used a combination of search terms for TB and implementation strategies, developed in collaboration with a Cochrane Infectious Diseases Group Information Specialist (see online supplemental appendix). We searched the following databases: MEDLINE (Ovid); Embase (Ovid); Cumulative Index to Nursing and Allied Health Literature (EBSCOHost); PsycInfo (EBSCOHost); Web of Science; WHO International Clinical Trials Registry Platform on 29 July 2022, regardless of language. We limited all searches to 2010 onwards because the development of Xpert MTB/RIF was completed in 2009. We contacted researchers and checked the references of relevant systematic reviews to identify any additional eligible studies. In addition, the WHO issued a public call for evidence, including operational reports.

### Selection of studies and data extraction

We used Covidence to manage the selection of studies.^15^ Four review authors (AP, MOB, ME and BV) independently and in parallel scrutinised all titles and abstracts and reviewed all full texts to determine studies for inclusion and reasons for exclusion. All disagreements were resolved by discussion with assistance from other review authors (RRN and/or KS).

We categorised eligible studies into high-thickness, medium-thickness or thin studies. High-thickness studies were classified as such if the authors described or evaluated an mWRD implementation strategy and (a) analysed their findings beyond a descriptive list of barriers and enablers, (b) demonstrated participants’ perspectives and experiences, (c) explained variation and illustrated meaning and (d) developed or contributed to broader understanding of mWRD implementation.^10 16^ Studies of medium-thickness met one or two but not all of these criteria, and thin studies did not demonstrate any of these criteria despite yielding some relevant insight on mWRD implementation. We prioritised data extraction and analysis of the high-thickness and medium-thickness studies and then revisited the thin studies for additional or contradictory insights.

Study findings were extracted in narrative form in Microsoft Word. For all studies, we extracted data that indicated the circumstances for mWRD testing and details on the population and health setting in which the test was evaluated, along with factors affecting the use of the test. If available, we also extracted data on factors that we expected to be important to user experiences, considering: what added value to the particular user, workflow, resources involved in implementing the test, confidence in test results, implementation process and concerns for access and equity. For studies with qualitative evidence, we extracted data on themes, categories, findings, supporting quotations and conclusions. We also recorded intervention design and qualitative assessment of effectiveness, barriers and enablers, and test or intervention costs. For studies with quantitative evidence, we included data that indicated whether the use or implementation of an mWRD or mWRD implementation strategy in the given context was effective. We did not perform a meta-analysis of summary quantitative outcomes, given the heterogeneity of study designs and outcomes.

Pairs of review authors extracted data independently from high-thickness studies (MOB, AP, ME, HMvdW, RRN) and generated memos for each study. Any conflicts were reconciled by consensus, including other senior authors (AMD, KS). Authors of primary studies did not extract data from their own study or studies.

### Quality assessment

Three review authors (AP, KS and RRN) working as pairs independently appraised study quality (see online supplemental table S6) using an assessment tool adapted from Standards for Reporting Implementation Studies (StaRI) statement.^17^ The StaRI statement encourages researchers to record their implementation strategy and the effectiveness of the intervention. As such, StaRI is mainly a reporting mechanism. Our interest was quality assessment. Using six domains that incorporated the core StaRI checklist items, we appraised studies according to study rationale, design, description of the implementation strategy, analytical rigour, interpretation of findings and policy implications. While studies were not given overall quality ratings, we assessed quality of each domain based on breadth and depth of the description of the strategy and reporting of implementation design and evaluation to inform stakeholders seeking to implement or adapt these types of strategies. We resolved disagreements by consensus discussion.

### Data synthesis

Our analyses focused on the high-thickness studies, which evaluated or discussed mWRD implementation strategies. We analysed medium-thickness studies for data on barriers and enablers and thin studies for additional insights that complemented or refuted our primary study findings. We categorised enablers and barriers—forces or factors that facilitated or enhanced implementation versus those that complicated or restricted mWRD use—at patient, provider and health system levels.

We used a thematic approach to guide data analysis.^18^ We developed initial themes based on a framework organised according to steps of the TB cascade of care and the data collected. First, we synthesised the findings from quantitative, qualitative and mixed methods studies to understand mWRD implementation strategies and their associated barriers and enablers, from a wide range of user perspectives. Next, we classified implementation strategies into nine broad categories modified from those defined by a key implementation science stakeholder concept mapping exercise conducted through the Expert Recommendations for Implementing Change study.^19^ These were: engaging stakeholders, using evaluative and iterative strategies, changing infrastructure, adapting and tailoring to the context, developing stakeholder inter-relationships, using financial strategies, supporting clinicians, providing interactive assistance and educating and training stakeholders. Finally, we used this framework to present implementation strategies alongside their core components and key implementation outcomes, for example, feasibility, acceptability and equity, to facilitate decision making by implementers and policymakers.

### Patient and public involvement

Patients or the public were not involved in the design, conduct, reporting of our systematic review and meta-analysis. However, many contributing studies did involve patients and community stakeholders in the design and reporting of their study results.

## Results

**Figure 1:**
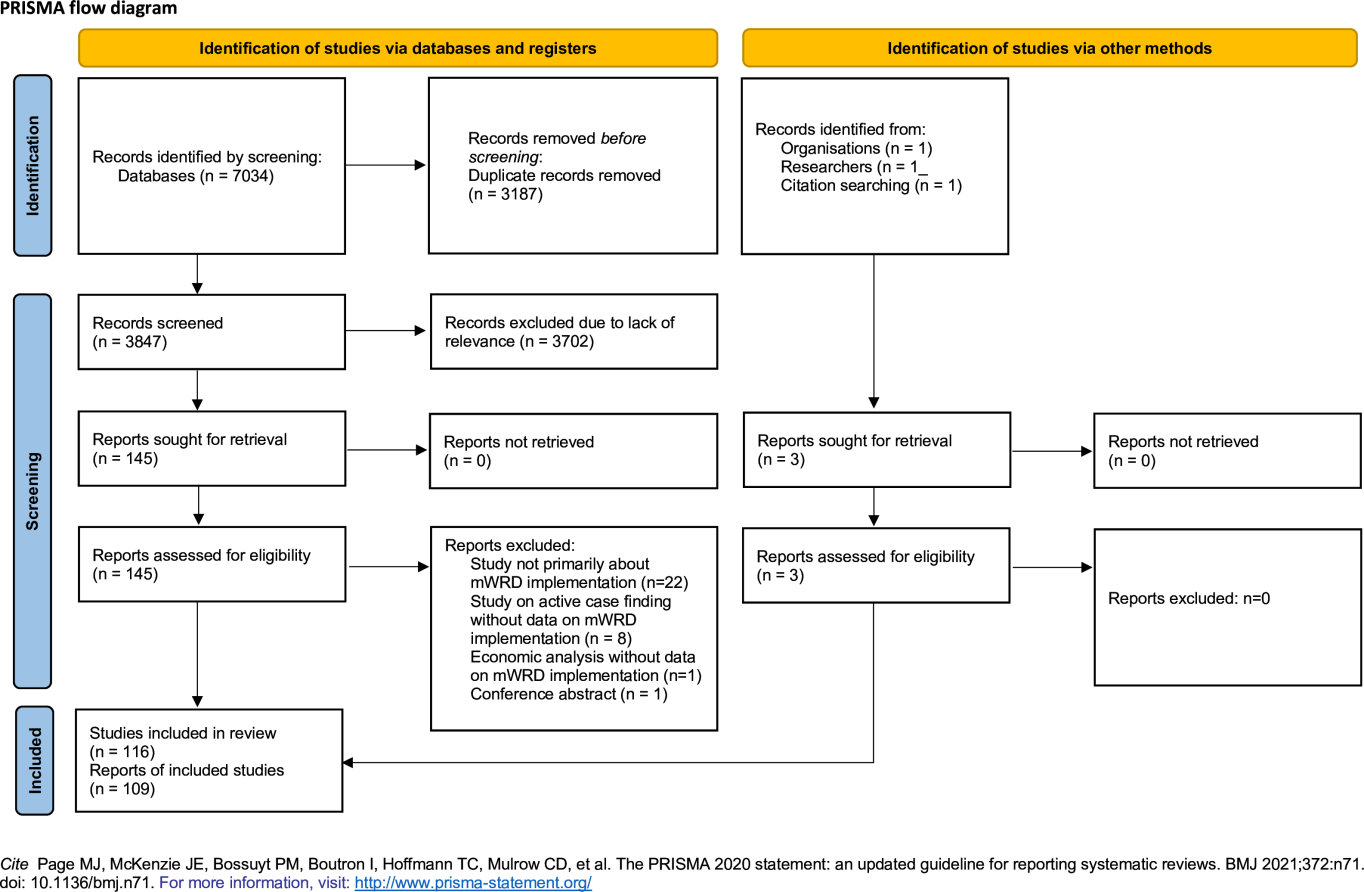
Preferred Reporting Items for Systematic Reviews and Meta-Analyses flow diagram. mWRD, molecular WHO-recommended rapid diagnostic test.

Our search yielded 7034 records; after removal of duplicates, we screened 3847 abstracts (figure 1). We included 109 studies (after merging seven reports about the same implementation strategy) and classified 54 studies as high-thickness (onlinesupplemental table S1S2), 41 as medium-thickness (online supplemental table S3) and 14 as thin (online supplemental table S4). High-thickness studies were conducted in 18 countries across all six WHO geographic regions (online supplemental figure S1) and used the following study designs: randomised controlled trials, quasi-experimental interventions, qualitative, mixed-methods, operational studies (majority) and systematic reviews. Operational study designs included: pre-post studies, single arm interventional pilots and policy transfer analysis. Operational reports were also reviewed for additional insights (online supplemental table S5).

### mWRD study quality assessment

Overall, the high-thickness studies provided both breadth and depth regarding implementation context and the implementation strategy being evaluated (see online supplemental table S6). However, few studies rigorously reported key implementation design and outcome components, which limit assessment of reproducibility and applicability of findings to other settings.

### Barriers and enablers to mWRD use and effect

Patient-level barriers included direct and indirect costs, gaps in individual and community education and awareness, health system mistrust and TB-related stigma that affected health-seeking behaviour including accessing diagnostic testing.^20^,^23^ Patient-level enablers included approaches to improve access to mWRD testing in peripheral healthcare settings, support for direct and indirect costs and stigma mitigation interventions.^21 24^

**Figure 2:**
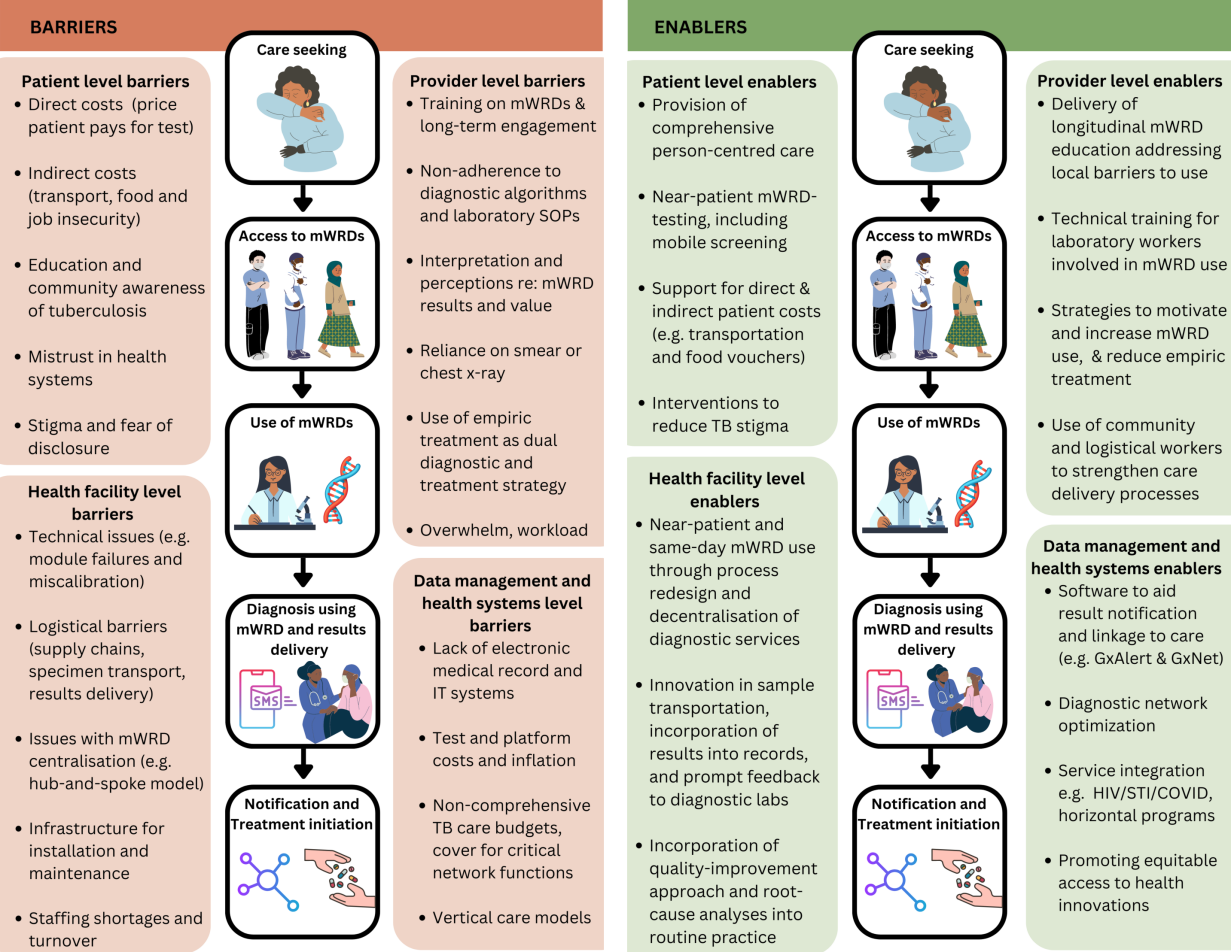
mWRD implementation barriers and enablers. mWRD, molecular WHO-recommended rapid diagnostic test; SOP, standard operating procedure; STI, sexually transmitted infection.

Reported provider-level barriers included gaps in training related to the utility and interpretation of mWRDs, as well as technical processes that can cause invalid results^25^,^30^ and health worker capacity.^31^,^33^ Provider-level enablers include delivery of high-quality training and longitudinal health worker engagement across the cascade of care and strategic use of support workers to close access and engagement gaps.^2734^,^37^

Health facility-level barriers included technical issues, such as module failures and lack of calibration and logistical and infrastructural barriers, including supply chain challenges like stock-outs, sample transportation, results delivery, reliance on centralised testing and staffing.^38^,^42^ Health facility-level enablers included same-day testing using process innovation and quality improvement strategies and innovations for sample transport and linkage.^43^,^48^

Health systems and data management-level barriers included the lack of electronic record systems and information technology software and prohibitive costs of tests and platforms for TB as a vertical (also referred to as stand-alone) disease programme.^4649^,^52^ Health systems and data management-level enablers included diagnostic network improvement, service integration and software to streamline notification and linkage.^2253^,^59^ Figure 2 summarises barriers and enablers across multiple levels.

### Strategies used to enable mWRD implementation across the diagnostic cascade

#### Step 1: detecting presumptive TB

**Figure 3:**
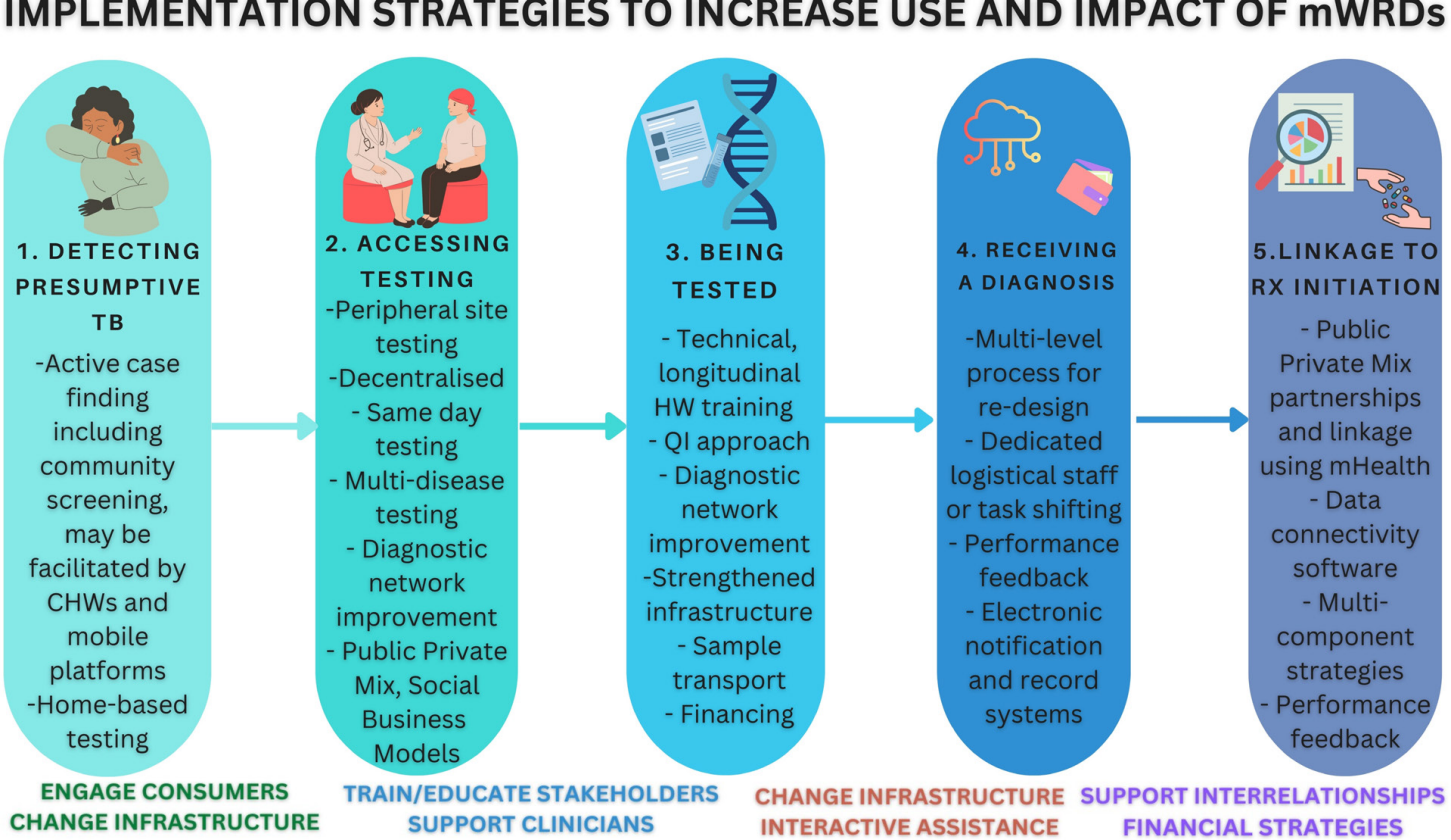
mWRD implementation strategies. mWRD, molecular WHO-recommended rapid diagnostic test.

Implementation strategies included engaging patients as key stakeholders in TB care, educating and training patients as stakeholders, developing stakeholder inter-relationships and changing infrastructure. Examples included mobile deployment of Xpert to test mining and other key communities at a public event in South Africa^60^ and combining Xpert with digital chest X-ray (dCXR) screening with computer automated detection to screen high-risk communities in Lima, Peru,^61^ along with home-based testing using a modified GeneXpert platform in South Africa to expand diagnostic outreach efforts which promoted trust.^62^ These strategies expand reach (step 1) and access (step 2) (figure 3).

#### Step 2: accessing testing

Implementation strategies to address facility-level barriers included changing infrastructure to conduct mWRDs in peripheral healthcare settings, which required mitigation of barriers like human resources constraints, inadequate cartridge supplies, module failure and inconsistent power supply, optimising sample transportation systems using existing hub-and-spoke model or clinic process redesign to improve efficiency and reduce delays, including multidisease testing platforms.^63^,^66^ Pooled testing can also enable larger scale screening of at-risk populations.^67^ Other strategies adapted mWRD use to facilitate private sector testing, including social enterprise models.^68 69^ Several studies used Xpert for follow-on testing after dCXR^70 71^ or symptom screening, including in prisons. This highlighted the importance of considering equity to expand test access and use in high-risk and often marginalised populations.^6672^,^75^

#### Step 3: being tested

Implementation strategies included engaging clinicians as users, along with education, training and support to improve the likelihood of health workers using mWRDs and reducing reliance on smear microscopy and empiric treatment.^76^,^79^ Strategies addressed facility-level and systems-level barriers by developing public and private sector relationships, including the use of public private mix or social business models^76 80 81^ and iterative strategies to redesign clinic, laboratory and pharmacy workflows incorporating quality improvement performance feedback.^5564 82^,^86^ Many studies emphasised multicomponent strategies that target multiple barriers associated with diagnosis and linkage to treatment initiation. Efforts to re-design processes and workflow at different levels, including the clinic, laboratory and pharmacy, increased Xpert use in clinics in Uganda, when coupled with performance feedback on cascade indicator metrics.^87^,^90^ Prioritising mWRD testing within facility-based TB transmission prevention strategies such as Find cases Actively, Separate temporarily and Treat effectively can shorten time-to-diagnosis and treatment but access and human resource barriers remain.^91 92^ Xpert evaluations revealed underutilisation due to the lack of regular maintenance of testing platforms, gaps in coordination to integrate mWRD into operational processes and between partners, including donors.^93^,^95^ Diagnostic network improvement, ideally done longitudinally and iteratively, can inform where and why mWRD underutilisation occurs at the regional or national level and can guide mitigation strategies.^63 71^

#### Step 4: receiving a diagnosis

Implementation strategies to address patient-level, provider-level, facility-level and data-management barriers included engaging patients and clinicians as users of mWRD and TB care data, changing infrastructure including electronic data systems, adapting and tailoring mWRD results delivery to the context and supporting clinicians. Examples included private sector provider mapping and longitudinal engagement complemented by Xpert testing offered free at the point of care, rapid specimen transport and high laboratory throughput, short message service (SMS) notifications to patients and clinicians^87 96 97^ and auxiliary workers alongside 24-hour Xpert testing.^98^

#### Step 5: linkage to timely treatment initiation

Implementation strategies optimised infrastructure using electronic data systems and mHealth technologies such as GxAlert (SystemOne, Boston, Massachusetts, USA), a cloud-based data monitoring and notification system that enables automatic results delivery to facilitate linkage.^99^,^101^ Persistent barriers included the lack of electronic record systems, software system interoperability and connectivity.^102 103^ Technical assistance, clinician support and evaluative strategies, including performance feedback, can improve treatment initiation.^104 105^ Developing financial strategies and public and private sector interrelationships enabled earlier treatment.^106^

### Key review findings

We synthesised our findings on implementation strategies across cascade steps into six key findings that can be used to inform mWRD and other diagnostic test implementation planning. Table 1 provides an evidence summary of these findings including the level of confidence in each finding (high confidence for three and moderate confidence for three).

**Table 1:** Summary of key findings of evidence synthesis with CERQual assessment

Key review finding	CERQual assessment of confidence in the evidence	Explanation of CERQual assessment	Studies contributing to review finding
Multicomponent strategies should address implementation barriers across the care cascade.	High confidence	We had no concerns about coherence, adequacy or relevance and only minor concerns about methodological quality of some included studies. Studies were from five different countries.	Cowan 2015, Manabe 2015, Schumacher 2015, Shete 2017, Awan 2018, Banu 2020, Cattamanchi 2020, Nalugwa 2020, Cattamanchi 2021, Reza 2021, Nalugwa 2022, Zawedde-Muyanja 2022
Person-centred approaches including community-based testing are needed to reach people with presumptive TB.	Moderate confidence	We had no concerns about coherence but had minor concerns about methodological quality of some included studies, adequacy due to small study numbers and relevance due to limited study/country settings. Studies were from two different countries.	Page-Shipp 2014, Zishiri 2015, Medina-Marino 2021, Yuen 2021
Innovation in diagnostic technology and implementation can support access to mWRDs in peripheral healthcare settings.	High confidence	We had no concerns about coherence, adequacy or relevance and only minor concerns about methodological quality of some included studies. Studies were from 12 different countries.	Clouse 2012, Durovni 2014, Theron 2014, Abdurrahman 2015, Schumacher 2015, Van den Handel 2015, Cowan 2016, Hanrahan 2016, Agizew 2017, Lessels 2017, Awan 2018, Stime 2018, Raizada 2018, Ngwira 2019, Jeyashree 2020, Cattamanchi 2021
Health worker training and engagement and diagnostic network improvement can increase the use of mWRDs as the first test for TB.	High confidence	We had no concerns about coherence, adequacy or relevance and only minor concerns about methodological quality of some included studies. Studies were from 18 different countries.	Creswell 2014, Colvin 2015, Manabe 2015, Pho 2015, Umubyeyi 2016, Nathavitharana 2017, Awan 2018, Gidado 2018, McDowell 2018, Ndlovu 2018, Raizada 2018, Stime 2018, Dabas 2019, Albert 2020, Banu 2020, Cattamanchi 2020, Deo 2020, Shibu 2020, Cattamanchi 2021, Deo 2021, Paudel 2021, Zawedde-Muyanja 2022
Improved data management systems can support linkage between diagnosis using a mWRD and delivery of results.	Moderate confidence	We had no concerns about coherence but had minor concerns about methodological quality of some included studies, adequacy due to small study numbers and relevance due to limited study/country settings. Studies were from four different countries.	Cowan 2016, Nathavitharana 2017, Raizada 2018, Babirye 2019
mHealth technologies can strengthen systems to support timely notification and treatment initiation.	Moderate confidence	We had no concerns about coherence but had minor concerns about methodological quality of some included studies and adequacy due to small study numbers. Studies were from five different countries.	Cowan 2016, Alagna 2020, Cattamanchi 2021, Khushvaktov 2021, Vatsyayan 2022, Zawedde-Muyanja 2022

mWRD, molecular WHO-recommended rapid diagnostic test; TB, tuberculosis.

*Multicomponent strategies should be designed and tested to address implementation barriers at all levels of the tuberculosis cascade*.^6468 69 82^,^84 87^ These strategies should be iteratively adapted with feedback obtained from robust quality improvement processes and operational research that considers contextual realities prior to and during implementation.

*Reaching all people with presumptive* TB requires a person-centred approach to TB care delivery that addresses the varied and complex barriers impacting engagement with TB diagnostic processes.^60^,^6275^ mWRD implementation strategies should include active case finding and community-based testing.

*Supporting access to mWRDs in peripheral healthcare settings* involves innovation in implementation including critical steps like sample transport to minimise delays.^64^,^6872^ Collaboration between private and public partners can shift mWRDs closer to where patients access care. Prioritising underserved and high-risk populations is critical to ensure equitable access to mWRD technologies.

*Ensuring that mWRDs are used as* *the* *first test for TB* requires high-quality training, collaboration and longitudinal engagement with health workers and other key implementers in public and private sectors.^5563 68 69 71 73 76 79^,^82 84 87 88 91^ Diagnostic network improvement should be undertaken periodically to ensure testing platforms are placed and used to address regional and national testing needs. Multidisease testing approaches should be implemented as part of broader health system strengthening efforts.

*Ensuring action following diagnosis using mWRD* is critical but often overlooked.^91 97 99 108^ Data management systems should be linked with electronic medical record systems and leverage SMS and other mHealth technologies including automatic electronic notifications.

*Developing systems to support timely notification and treatment initiation* should leverage mHealth solutions including software linked to mWRD platforms that facilitate linkage and retention throughout the cascade of care beyond diagnosis.^78 82 88 99 100 102^

## Discussion

This mixed-methods systematic review identified barriers and enablers to mWRD use and implementation and synthesised findings on implementation strategies that were used across programme contexts. While context contributes to implementation success in different settings, there are clear commonalities among barriers, enablers and strategies for mWRD implementation. These include the need to expand mWRD access and uptake in community and peripheral healthcare settings through approaches that align with person-centred care and leverage multicomponent strategies that optimise clinical and laboratory processes and use iterative performance feedback, multidisease testing platforms, diagnostic network improvement, coupled with digital and mobile health technologies to ensure prompt result notification and treatment initiation.

The central importance of multicomponent strategies to address patient, provider, health facility and systems level barriers^87 88 90^ was clear in most included studies, although few rigorously designed and implemented such strategies. Multicomponent strategies enable patients to benefit from innovative molecular diagnostic technologies^45^ by incorporating innovation in implementation processes such as sample transportation to enable testing in peripheral healthcare settings.^88^ Use of iterative feedback on testing process indicators supported by robust quality improvement processes can maximise impact.^64 87^

Our review highlights how expanding mWRD use is closely aligned with improving person-centred care that addresses the complex barriers faced by people with TB.^1^ This requires improved individual and community education awareness and efforts to reduce stigma and address intersecting health disparities that affect individuals and communities, including gaps in access to healthcare and transportation. These impacts are compounded in underserved populations who are often considered as being ‘hard to reach’,^21 78^ underscoring the need for implementation to centre equity. This includes closing care-seeking gaps with support from peer navigators and community health workers, improving test accessibility and facilitating results notification, which requires attention to digital health equity.^10^

High-quality, regular, longitudinal education and training with performance feedback for all health workers including laboratory staff and other key implementers involved in TB care^77^ is critical to strengthen mWRD implementation. Broader efforts to strengthen clinic, laboratory and pharmacy workflows are needed, ideally with process redesign and task shifting using additional support workers and minimal reliance on volunteers, especially in the context of overwhelmed health systems.^109^ Improved service integration such as multidisease platforms that enable diagnosis of other conditions such as HIV or SARS-CoV-2^23^ can optimise test throughput and efficiency and allow procurement and maintenance costs to be shared between programmes. This requires collaboration and clear division of responsibilities for supplies and maintenance. Health system strengthening is essential and can mitigate potential risks like reducing TB testing to enable SARS-CoV-2 testing, as seen during the COVID-19 pandemic. Improved integration between public and private sectors can support patient choices about healthcare engagement and can be aided by fostering longitudinal interrelationships, with interactive technical assistance and digital technologies. Potential tensions include consideration of profit incentives and patient trust in public versus private healthcare.

Diagnostic network improvement, repeated periodically at the national and regional level, can maximise the impact of mWRDs by identifying settings where decentralised testing can be most helpful and addressing inequitable geographic distribution of existing tests.^57 63 104^ There may be tensions between maximising diagnostic yield by concentrating testing infrastructure on high incidence settings versus equity by increasing testing in rural areas that may have lower incidence. Despite the explosion in mHealth technologies, including data connectivity software developed to strengthen mWRD results linkages, barriers including access to mobile phones, prohibitive data costs and power cuts, as well as health systems mistrust, may preclude patients benefiting from these technological advances.^97 99^ Donor dependency remains a common challenge to maintain subsidised test costs and expand services; this necessitates intersectoral resource mobilisation and continued advocacy.^63 93^

Strengths of our review include comprehensively searching numerous databases, handsearching references of other systematic reviews, contacting experts and a public call for additional evidence, including operational reports. There were limited data on mWRDs other than Xpert, and certain country contexts like South Africa, Uganda and India were disproportionately represented in included studies due to factors including rapid expansion of Xpert or existing research and organisational partnerships in these settings. While our search was not restricted by language, we did not identify studies for inclusion that were in languages other than English.

Similar to Brown *et al*,^11^ our review identified the limited use of implementation science frameworks and reporting metrics, aside from a few studies that used a rigorous implementation science approach to design and implement multicomponent strategies.^8283 87^,^89 95^ Standardised reporting using guidance from StARI^17^ can enhance assessment of implementation effectiveness and optimisation based on a contextual analysis of barriers and enablers that may be setting-specific. Improved implementation science capacity, including operational research, can bridge the implementation gap between translating research advances into policy and practice. We note that data on the cost-effectiveness of implementation strategies are of importance to implementers and programmes. The aim of this review was an in-depth analysis of the effectiveness of different implementation strategies to increase the impact of mWRD testing. Prior studies have evaluated the cost-effectiveness of Xpert for TB diagnosis.^110^,^112^ The example of decentralised Xpert testing using a multicomponent strategy that increased TB diagnoses showed similar per-test costs with modest incremental costs per diagnosis and treatment initiation versus Xpert testing at an off-site facility, suggesting such strategies can be cost-effective.^113^

## Conclusions

Our findings reveal the importance of multicomponent strategies and longitudinal training of health workers across the diagnostic care cascade to address patient, provider, health facility, data management and health systems-level barriers to the delivery of high-quality, person-centred TB care. Development of electronic record systems, aligned with specific software focused on TB results delivery and linkage, should be an urgent priority. Diagnostic network improvement should be undertaken longitudinally and repeatedly to optimise mWRD impact and programme decision making must centre on equity considerations. Adequate resource mobilisation and efforts to reduce donor dependencies remain fundamental challenges. Programmatic and policy decision makers should prioritise innovation in implementation processes to maximise the equitable impact of innovative technologies like mWRDs to realise strategic targets to end the TB pandemic.

## Supplementary material

10.1136/bmjgh-2024-018700 online supplemental file 1

10.1136/bmjgh-2024-018700 online supplemental file 2

10.1136/bmjgh-2024-018700 online supplemental file 3

10.1136/bmjgh-2024-018700 online supplemental file 4

10.1136/bmjgh-2024-018700 online supplemental file 5

10.1136/bmjgh-2024-018700 online supplemental file 6

10.1136/bmjgh-2024-018700 online supplemental file 7

10.1136/bmjgh-2024-018700 online supplemental file 8

## Data Availability

All data relevant to the study are included in the article or uploaded as supplementary information.
