# Supplementary material for: Implementation strategies to increase the uptake and impact of molecular WHO-recommended rapid diagnostic tests: evidence from a mixed-methods systematic review
Source: BMJ Glob Health. 2025 Sep 17;10(9):e018700. doi: 10.1136/bmjgh-2024-018700 (PMC12458786; doi:10.1136/bmjgh-2024-018700)
Supplement: online supplemental file 1 [file bmjgh-10-9-s001.pdf]

APPENDIX

Review questions

What barriers and enablers affect the use or impact of molecular WHO-recommended rapid diagnostic tests for the detection of tuberculosis and tuberculosis drug resistance?

How have implementation strategies successfully addressed barriers to the use or impact of molecular WHO-recommended rapid diagnostic tests for the detection of tuberculosis and tuberculosis drug resistance?

How do implementation strategies improving the use or impact of molecular WHO-recommended rapid diagnostic tests for the detection of tuberculosis and tuberculosis drug resistance affect the following outcomes:

- implementation outcomes: acceptability, adoption, appropriateness, costs, feasibility, fidelity, penetration, and sustainability

- service or system outcomes: efficiency, safety, effectiveness, equity, patient-centeredness, timeliness

- user outcomes: satisfaction, function, symptomatology, stigma

Definitions

Intervention: The process of intervening on people, groups, entities or objects in an experimental study, programmatic, or policy setting[1110110110110]. Depending on the study, programme, or policy design, an intervention may be compared to a control, which may be placebo or an alternative intervention in the comparison groups. Interventions can be used as in their own right or can be used as an implementation strategy.

Implementation strategies: ‘Methods or techniques used to enhance the adoption, implementation, and sustainability of a clinical programme or practice’, which in the case of this review is molecular WHO-recommended rapid diagnostic tests. Intervention strategies may be single-component, for example, placement of the test in a given setting, or dissemination of guidelines that outline testing recommendations, or multi-component, for example, a staged approach that moves from training to consultation to audit and feedback, or one that includes distinct intervention components that are combined in recognition of the contextual factors in a given health system or setting that affects test implementation.

Barrier: A systemic and/or experienced force or factor that reduces or removes the potential intended patient- or population-level effects of molecular WHO-recommended rapid diagnostic tests.

Enabler: A systemic and/or experienced factor or force that enhances or achieves the potential intended patient- or population-level effects of molecular WHO-recommended rapid diagnostic tests.

Context: reflects a set of characteristics and circumstances that consist of active and unique factors, within which the implementation is embedded. As such, context is not [just] a backdrop for implementation, but interacts, influences, modifies and facilitates or constrains the intervention and its

implementation. Context is usually considered in relation to an intervention, with which it actively interacts. It is an overarching concept, comprising not only a physical location but also roles, interactions and relationships at multiple levels.

Use: The processes whereby molecular WHO-recommended rapid diagnostic tests are utilized and incorporated into tuberculosis care delivery systems at the level of the user (to include patient, clinician, laboratory staff), health system, or policymakers.

Impact: 'The extent to which an intervention has generated or is expected to generate significant positive or negative, intended or unintended, higher-level effects'[2111111111111]. Impact addresses the ultimate significance and potentially transformative effects of the intervention. It seeks to identify social, environmental and economic effects of the intervention that are longer term or broader in scope than those already captured under the effectiveness criterion. Beyond the immediate results, this criterion seeks to capture the indirect, secondary and potential consequences of the intervention. It does so by examining the holistic and enduring changes in systems or norms, and potential effects on people's well-being, human rights, gender equality, and the environment.'

Implementation success: The evaluation of implementation success will consist of an assessment of a series of outcomes that we have adapted from Proctor et al[3]. These will include implementation outcomes (acceptability, adoption, appropriateness, costs, feasibility, fidelity, penetration, and sustainability), service outcomes (efficiency, safety, effectiveness, equity, patient-centeredness, timeliness), and user outcomes (satisfaction, function, symptomatology, and stigma). We note successful implementation varies by setting, for example, mWRD rapid diagnostic test use may not always diagnose more people but may do so faster or improve access to initial testing for drug resistant tuberculosis.

Inclusion criteria

Study design types

Qualitative study designs included ethnography, phenomenology, case studies, grounded theory studies, and qualitative process evaluations. Qualitative studies were included if they used qualitative methods for data collection (such as in-depth interviews or group discussions) and qualitative data analysis methods. Quantitative study designs included intervention trials, cohort studies, pre- and post-studies or interrupted time series. We included these types of studies to obtain data regarding barriers, enablers, and implementation approaches (how to), rather than to assess the effectiveness or accuracy of mWRDs. We included systematic reviews related to our topic of interest. Unless, they had data on barriers, enablers, or implementation strategies, we excluded studies on active case finding and economic analyses.

mWRDs included in the review

We included studies that evaluated implementation strategies or interventions that included the following mWRDs: Xpert MTB/RIF, Xpert MTB/RIF Ultra, Xpert MTB/XDR, Xpert Edge, Truenat MTB, Truenat MTB Plus, Truenat MTB-Rif Dx, TB-LAMP, and moderate complexity automated nucleic acid amplification tests (RealTime MTB RIF/INH, BD MAX™ MDR-TB, cobas® MTB-RIF/INH, and FluoroType® MTBDR).

Table S1. Characteristics of studies of mWRD implementation, high thickness studies

Table S2. Additional characteristics of studies of mWRD implementation, high thickness studies

Table S3. Characteristics of studies of mWRD implementation, medium thickness studies

Table S4. Characteristics of studies of mWRD implementation, thin studies

Table S5. Characteristics of operational reports with data on mWRD implementation strategies

Table S6. Assessment of methodological limitations using an adapted version of the Standards for Reporting Implementation Studies (StaRI).

Figure S1. Countries represented across all six WHO geographic regions in high-thickness studies.

Ovid MEDLINE(R) and In-Process, In-Data-Review & Other Non-Indexed Citations <1946 to July 26, 2022>

1	exp Tuberculosis/	202469
2	*Mycobacterium tuberculosis/	41074
3	tuberculosis.ti,ab. or (MDR-TB or XDR-TB).tw.	203786
4	1 or 2 or 3	266677
5	(Truenat* or Molbio).mp.	45
6	(Genexpert* or Xpert* or MTB*RIF or Xpert ultra).mp.	3552
7	(Xpert* adj2 TB).tw.	147
8	*Point-of-Care Systems/	8963
9	(drug susceptibility test* or drug resistance test* or (rapid adj3 (detect* or test* or diagnos*)) or (poc or poct or "point of care")).tw.	95707
10	(cartridge adj2 (test* or assay*)).tw.	133
11	TB-LAMP.mp.	36
12	loop-mediated isothermal amplification.mp.	3855
13	Nucleic Acid Amplification Techniques/ or NAAT*.mp.	13863
14	MTB-RIF*INH.tw.	0
15	5 or 6 or 7 or 8 or 9 or 10 or 11 or 12 or 13 or 14	112450
16	4 and 15	8386
17	limit 16 to yr="2010 -Current"	6268
18	*implementation science/	587
19	Health Plan Implementation/	6627
20	(implementation or implemented).ti,ab.	478021
21	"Patient Acceptance of Health Care"/ or acceptability.tw.	94412
22	(adoption or adopted).tw. not Adoption/	183242
23	(Appropriateness or feasibility or readiness or penetration or reach).tw.	511184
24	*Program Evaluation/	11476
25	Sustainability.tw.	32087
26	patient satisfaction/ or patient preference/	97714
27	*"Attitude of Health Personnel"/	65936
28	patient retention.tw. or Patient Compliance/	60634

29 (facilitat* or barrier* or challeng* or "process analysis" or enabl* or "change agent*").tw. 2322253

30 *Motivation/ 31851

31 (perception* or experience*).tw. 1465795

32 exp Health Equity/ 2889

33 cost effectiveness.mp. or Cost-Benefit Analysis/ 121713

34 Health Care Costs/ or Health Expenditures/ 63877

35 18 or 19 or 20 or 21 or 22 or 23 or 24 or 25 or 26 or 27 or 28 or 29 or 30 or 31 or 32 or 33 or 34 4723164

36 17 and 35 2042

37 qualitative.tw. or Qualitative Research/ 295677

38 mixed method*.tw. 32237

39 Interviews as Topic/ or interview*.mp. 446413

40 "Surveys and Questionnaires"/ or survey*.tw. 1121031

41 (ethnograph* or phenomenolog*).tw. 42274

42 *grounded theory/ 229

43 exp Focus Groups/ 34483

44 framework analysis.tw. 2546

45 37 or 38 or 39 or 40 or 41 or 42 or 43 or 44 1647261

46 17 and 45 409

47 36 or 46 2241

Embase 1947-Present, updated daily

1 exp Tuberculosis/ 285972

2 *Mycobacterium tuberculosis/ 32163

3 tuberculosis.ti,ab. or (MDR-TB or XDR-TB).tw. 248607

4 1 or 2 or 3 342348

5 (Truenat* or Molbio).mp. 102

6 (Genexpert* or Xpert* or MTB*RIF or Xpert ultra).mp. 7492

7 (Xpert* adj2 TB).tw. 221

8 "*Point-of-Care System"/ 0

9 (drug susceptibility test* or drug resistance test* or (rapid adj3 (detect* or test* or diagnos*)) or (poc or poct or "point of care")).tw. 126872

10 (cartridge adj2 (test* or assay*)).tw. 268

11 TB-LAMP.mp. 46

12 loop-mediated isothermal amplification.mp. 5487

13 Nucleic Acid Amplification Techniques/ or NAAT*.mp. 2801

14 MTB-RIF*INH.tw. 0

15 5 or 6 or 7 or 8 or 9 or 10 or 11 or 12 or 13 or 14 137418

16 4 and 15 11562

17 limit 16 to yr="2010 -Current" 9285

18 *implementation science/ 1266

19 Health Plan Implementation/ 105965

20 (implementation or implemented).ti,ab. 647622

21 patient attitude/ or acceptability.tw. 131344

22 (adoption or adopted).tw. not Adoption/ 231150

23 (Appropriateness or feasibility or readiness or penetration or reach).tw. 719921

24 *Program Evaluation/ 2638

25 Sustainability.tw. 38767

26 patient satisfaction/ or patient preference/ 179915

27 *health personnel attitude/ 43898

28 patient retention.tw. or Patient Compliance/ 145603

29 (facilitat* or barrier* or challeng* or "process analysis" or enabl* or "change agent*").tw. 2957314

30 *Motivation/ 32918

31 (perception* or experience*).tw. 2123394

32 exp Health Equity/ 7046

33 cost effectiveness.mp. or Cost-Benefit Analysis/ 271136

34 Health Care Costs/ or "Health Expenditures".tw. 177624

35 18 or 19 or 20 or 21 or 22 or 23 or 24 or 25 or 26 or 27 or 28 or 29 or 30 or 31 or 32 or 33 or 34 6545430

36 17 and 35 3080

37 qualitative.tw. or Qualitative Research/ 380778

38 mixed method*.tw. 39428

39 Interview* tw.mp. [mp=title, abstract, heading word, drug trade name, original title, device manufacturer, drug manufacturer, device trade name, keyword heading word, floating subheading word, candidate term word] 0

40 (Survey* or questionnaire*).tw. 1718505

41 (ethnograph* or phenomenolog*).tw. 48719

42 *grounded theory/ 833

43 framework analysis.tw. 3392

44 37 or 38 or 39 or 40 or 41 or 42 or 43 2088122

45 17 and 44 566

46 36 or 45 3395

47 limit 46 to human 3056

48 limit 47 to conference abstracts 587

49 47 not 48 2469

Cinahl (EBscoHost)

Limiters - Published Date: 20100101-20221231

#	Query	Results
S21	S18 OR S20	309
S20	S9 AND S19	121
S19	TX (qualitative research or qualitative study or qualitative methods or interview) OR TX mixed methods OR TX (interview* or survey*) OR TX (ethnography or phenomenology or grounded theory) OR TX (focus group* or framework analysis)	840,418
S18	S10 AND S17	242
S17	S11 OR S12 OR S13 OR S14 OR S15 OR S16	927,057
S16	(cost effectiveness or cost benefit or economics or cost management or economics) OR MH Health Care Costs OR TX (health expenditure or health spending or health expenses)	214,723
S15	TI (Motivation or perception* or experience* or Equity) OR AB (Motivation or perception* or experience* or Equity)	466,225

S14	MH attitude of health personnel OR TI (facilitat* or barrier* or challeng* or "process analysis" or enabl* or "change agent*")) OR TI (facilitat* or barrier* or challeng* or "process analysis" or enabl* or "change agent*"))	102,796
S13	MH (program evaluation or program effectiveness or program assessment) OR TI (Sustainability or satisfaction or preference*) OR AB (Sustainability or satisfaction or preference*)	137,189
S12	MH patient acceptance of health care OR TI (Appropriateness or feasibility or readiness or penetration or reach) OR AB (Appropriateness or feasibility or readiness or penetration or reach)	99,824
S11	TX (implementation strategies or implementation methods) OR TX implemented OR MH Health Plan Implementation	67,039
S10	S3 AND S8	1,184
S9	S3 AND S8	1,350
S8	S4 OR S5 OR S6 OR S7	22,342
S7	TI (TB-LAMP or "loop-mediated isothermal amplification" or "Nucleic Acid Amplification Techniques" or NAAT*) OR AB (TB-LAMP or "loop-mediated isothermal amplification" or "Nucleic Acid Amplification Techniques" or NAAT*)	625
S6	TI (cartridge N2 (test* or assay*)) OR AB (cartridge N2 (test* or assay*))	57
S5	MH point of care systems OR TI ((drug susceptibility test* or drug resistance test* or (rapid N3 (detect* or test* or diagnos*) or (poc or poct or "point of care")) OR AB ((drug susceptibility test* or drug resistance test* or (rapid N3 (detect* or test* or diagnos*) or (poc or poct or "point of care"))	21,334
S4	TI ((Truenat* or Molbio) or (Genexpert* or Xpert* or MTB*RIF or Xpert ultra)) OR AB ((Truenat* or Molbio) or (Genexpert* or Xpert* or MTB*RIF or Xpert ultra))	762
S3	S1 OR S2	27,835
S2	TI ((MDR-TB or XDR-TB)) OR AB ((MDR-TB or XDR-TB))	934
S1	TI (tuberculosis or mycobacterium tuberculosis or tb) OR AB (tuberculosis or mycobacterium tuberculosis or tb)	27,835

PsycInfo (EBScoHost)

Limiters - Published Date: 20100101-20221231

#	Query	Results

S22	S18 OR S20	19
S21	S18 OR S20	20
S20	S9 AND S19	10
S19	TX (qualitative research or qualitative study or qualitative methods or interview) OR TX mixed methods OR TX (interview* or survey*) OR TX (ethnography or phenomenology or grounded theory) OR TX (focus group* or framework analysis)	1,100,207
S18	S10 AND S17	14
S17	S11 OR S12 OR S13 OR S14 OR S15 OR S16	862,135
S16	(cost effectiveness or cost benefit or economics or cost management or economics) OR Health Care Costs OR TX (health expenditure or health spending or health expenses)	133,644
S15	TI (Motivation or perception* or experience* or Equity) OR AB (Motivation or perception* or experience* or Equity)	574,842
S14	attitude of health personnel OR TI (facilitat* or barrier* or challeng* or "process analysis" or enabl* or "change agent*")) OR TI (facilitat* or barrier* or challeng* or "process analysis" or enabl* or "change agent*"))	55,947
S13	(program evaluation or program effectiveness or program assessment) OR TI (Sustainability or satisfaction or preference*) OR AB (Sustainability or satisfaction or preference*)	152,806
S12	patient acceptance of health care OR TI (Appropriateness or feasibility or readiness or penetration or reach) OR AB (Appropriateness or feasibility or readiness or penetration or reach)	68,187
S11	TX (implementation strategies or implementation methods) OR TX implemented OR Health Plan Implementation	49,376
S10	S3 AND S8	40
S9	S3 AND S8	49
S8	S4 OR S5 OR S6 OR S7	4,062
S7	TI (TB-LAMP or "loop-mediated isothermal amplification" or "Nucleic Acid Amplification Techniques" or NAAT*) OR AB (TB-LAMP or "loop-mediated isothermal amplification" or "Nucleic Acid Amplification Techniques" or NAAT*)	100
S6	TI (cartridge N2 (test* or assay*)) OR AB (cartridge N2 (test* or assay*))	2
S5	point of care systems OR TI ((drug susceptibility test* or drug resistance test* or (rapid N3 (detect* or test* or diagnos*) or (poc or poct or "point of care")) OR AB (	3,953

	(drug susceptibility test* or drug resistance test* or (rapid N3 (detect* or test* or diagnos*) or (poc or poct or "point of care"))	
S4	TI ((Truenat* or Molbio) or (Genexpert* or Xpert* or MTB*RIF or Xpert ultra)) OR AB ((Truenat* or Molbio) or (Genexpert* or Xpert* or MTB*RIF or Xpert ultra))	17
S3	S1 OR S2	3,269
S2	TI ((MDR-TB or XDR-TB)) OR AB ((MDR-TB or XDR-TB))	56
S1	TI (tuberculosis or mycobacterium tuberculosis or tb) OR AB (tuberculosis or mycobacterium tuberculosis or tb)	3,269

Web of Science Core Collection

Editions = CPCI-SSH , CPCI-S , SCI-EXPANDED , SSCI

#13 #10 OR #12

1,960

#12 #8 AND #11

408

Search

#11 qualitative research o "qualitative study" or "qualitative methods" or interview* OR "mixed methods" (Topic) or **(interview* or survey*)** (Topic) or **"grounded theory "** (Topic) or **ethnography or phenomenology** (Topic) or **"focus group*" or "framework analysis"** (Topic)

1,926,623

#10 #8 AND #9

2,827

#9 (implementation strategies or implementation methods) OR implemented (Topic) or **Appropriateness or feasibility or readiness or penetration or reach or acceptance** (Topic) or **"program evaluation" or "program effectiveness" or "program assessment" OR sustainability or satisfaction or preference*** (Topic) or **facilitat* or barrier* or challeng* or "process analysis" or enabl* or "change agent*"** (Topic) or **(Motivation or perception* or experience* or Equity)** (Topic) or **"cost effectiveness" or economics or "Health Care Costs "** (Topic)

10,246,698

#8 #6 AND #3 and **2022** or **2021** or **2020** or **2019** or **2018** or **2017** or **2016** or **2015** or **2014** or **2013** or **2012** or **2010** or **2011** (Publication Years)

8,556

#7 #6 AND #3

12,014

#6 #5 OR #4

426,939

#5 cartridge and (test* or assay*) (Topic) or **TB-LAMP or "loop-mediated isothermal amplification" or "Nucleic Acid Amplification Techniques" or NAAT*** (Topic) or **"drug susceptibility test*" or "drug resistance test*"** (Topic)

15,049

#4 (Truenat* or Molbio) or (Genexpert* or Xpert* or MTB*RIF or Xpert ultra) (Topic) or **"point of care systems"** (Topic) or **poc or poct or "point of care"** (Topic) or **"drug susceptibility test*" or "drug resistance test*"** (Topic) or **rapid and (detect* or test* or diagnos*)** (Topic)

420,184

#3 #1 OR #2

258,846

#2 MDR-TB or XDR-TB (Topic)

5,207

#1 (tuberculosis OR tb OR mycobacterium) (Topic)

258,846

WHO ICTRP

24 records for 24 trials found for: tuberculosis and diagnos* and (implement* or impact or qualitat* or survey or preference*)

Google Scholar

tuberculosis and rapid AND diagnos* and (implement * or preferences or qualitative)

2010-2022

Screened first 100 records

Implementation strategies to increase the uptake and impact of molecular WHO-recommended rapid diagnostic tests: Evidence from a mixed-methods systematic review.

Data extraction form

Instructions:
Save one document for each publication reviewed. The document should be saved as "First author last name Year _Covidence Number_Review author initials".
Example: "Engel 2015_1144_MOB"

General Information:
Systematic review team author:
Covidence Manuscript #:
First author, last name:
Title:
Year:
Name and email of corresponding author:
Language:

Research questions/objectives (free text box):

Study type
- Qualitative
- Quantitative
    - RCT
    - Cohort
    - Other (specify)
- Mixed methods

mWRD
- Xpert (specify MTB/RIF or Ultra)
- Truenat
- TB-LAMP
- Other (*specify*)

mWRD implementation strategy (*e.g. Xpert implemented at point-of-care in clinic) including role of the test (e.g. Xpert add-on after LAMP vs replacement*) (free text box):

Rationale (basis for mWRD intervention based on known barriers/enablers) (free text box):

Target population (free text box):

User/implementer
- Healthcare providers
- Patients
- Laboratory staff
- Clinic manager
- TB programme manager
- Policy maker
- Other (specify)

Country (plus add OECD income classification- high income, high middle, low middle, low)

Geographical setting
- Urban
- Rural
- Both urban and rural

Type of health facility (public or private)
- Clinic
- Hospital
- Laboratory
- Community health centre
- Mobile testing vehicle
- Other

- TB burden country (https://www.who.int/news/item/17-06-2021-who-releases-new-global-lists-of-high-burden-countries-for-tb-hiv-associated-tb-and-drug-resistant-tb)
    - High TB burden country yes, no
    - High TB/HIV burden yes, no
    - High MDR-burden yes, no

Context (*add any other considerations such as socio-cultural (comprises values, knowledge, beliefs, customs of a group), socio-economic (comprises social and economic resources), ethical (comprises norms, rules, standards of conduct)*) (text box):

Methods
Method of data collection:
Qualitative
- Survey
- Focus groups
- Key informant interviews
- Other (specify)

Quantitative
- Survey
- Case record forms
- Effectiveness outcome data
- Other (specify)

Data analysis methods (*mention if conceptual framework included):*
- Thematic analysis
- Framework analysis
- Narrative analysis
- Implementation framework such as RE-AIM or CFIR
- Other (specify, can list here if unsure)

mWRD barriers (free text box, clarify if these were addressed/identified in this study):

mWRD enablers (free text box, clarify if these were used/identified in this study):

Themes – highlight and write in free text relevant insights/quotes

Patient care related (drop down with list plus text box below)

- Care (model of care, person-centredness)
- Access
- Responsibility
- Education
- Stigma
- Support

Health systems related (drop down with list plus text box below)

- Files and records

- Integration of services
- Notifications
- Diagnostics – including laboratory infrastructure/strengthening
- Staffing - including training
- Messaging and media
- Outreach
- Readiness
- Electronic and digital systems

Outcomes (refer to Proctor 2011 Implementation Outcomes paper for definitions):

Implementation outcomes (drop down with list plus text box below):
- Acceptability
- Adoption
- Appropriateness
- Costs
- Feasibility
- Fidelity
- Penetration
- Sustainability

Service or system outcomes (drop down with list plus text box below):
- Efficiency
- Safety
- Effectiveness
- Equity
- Patient-centeredness
- Timeliness

User outcomes (drop down with list plus text box below):
- Satisfaction
- Function
- Symptomatology
- Stigma

Summary of Findings/Key *Qualitative* Message related to impact of implementation strategy/intervention on mWRD implementation/use/scale up:

