# Supplementary figures and images for: Implementation strategies to increase the uptake and impact of molecular WHO-recommended rapid diagnostic tests: evidence from a mixed-methods systematic review
Source: BMJ Glob Health. 2025 Sep 17;10(9):e018700. doi: 10.1136/bmjgh-2024-018700 (PMC12458786; doi:10.1136/bmjgh-2024-018700)

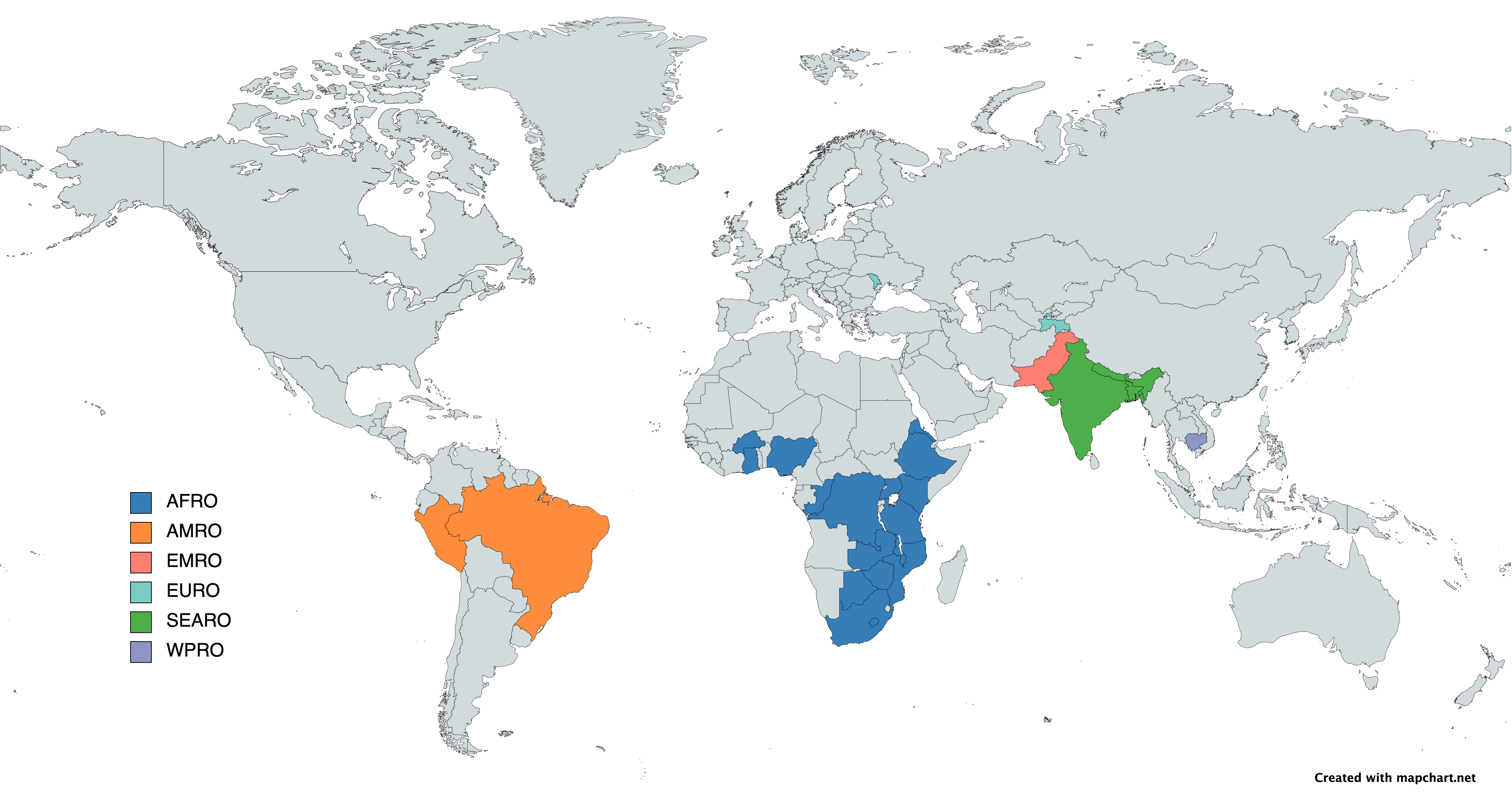

Supplement: online supplemental file 2 [file bmjgh-10-9-s002.png]
