# Supplementary material for: Implementation strategies to increase the uptake and impact of molecular WHO-recommended rapid diagnostic tests: evidence from a mixed-methods systematic review
Source: BMJ Glob Health. 2025 Sep 17;10(9):e018700. doi: 10.1136/bmjgh-2024-018700 (PMC12458786; doi:10.1136/bmjgh-2024-018700)
Supplement: online supplemental file 3 [file bmjgh-10-9-s003.docx]

| **Study and Country** | **mWRD implementation strategy** | **Population, setting** | **Design** | **Level of barriers addressed** | **Summary of how implementation strategy addressed barriers** | **Challenges identified** |
| --- | --- | --- | --- | --- | --- | --- |
| **Care seeking access** |  |  |  |  |  |  |
| Medina-Marino 2021  South Africa | Home-based testing using modified portable Xpert instrument (GX1) | Household TB contacts | Qualitative (nested within RCT) | Patient | Mobile mWRDs use enables home testing which addresses low referral rates and patient access barrier as well as engenders trust in process. | Time consuming  Concerns re: disclosure at home |
| Page-Shipp 2014  South Africa | POC Xpert at public event | Symptomatic patients from urban gold mining community, mobile testing vehicle | Operational research | Patient  Health systems | Use of mass community screening to test high-risk population | Need for back up electricity supply and web connectivity |
| **Access to mWRD** |  |  |  |  |  |  |
| Agizew 2017  Botswana | Centralized lab vs. POC Xpert | Patients at peripheral clinics | Stepped wedge trial | Health systems | Refresher training  Strengthening infrastructure  Supervisor support | Workload, staff turnover and training perceptions |
| Albert 2020  Lesotho | Diagnostic network optimization | Country wide | Operational research | Health systems | Relocating Xpert  Sample transport support  Integrated TB HIV care  POC platforms like Omni | Underutilization of Xpert, Sample transportation,  Linking people with symptoms and tests  Multiple partners/donors |
| Awan 2018  Pakistan | Multicomponent reverse public-private mix targeting public hospitals and PMDT sites and SBM targeting private sector | Presumptive TB patients, public and private sector facilities in urban city | Operational research: Cross-sectional intervention | Health systems | Use of community-based screeners and social business model using medical detailing and provision of free treatment | Equipment maintenance costs, supply chain issues, poor public-private integration |
| Banu 2020  Bangladesh | Multicomponent- 3 TB screening centres with free Xpert to which patients could be referred by public and private providers (latter engaged through social enterprise model) | Presumptive TB, urban | Operational research:  Cross-sectional intervention | Health systems  Providers | Subsidized digital CXR, Free Xpert  Radiography revenues supported screening centre costs  Patients could choose private vs public | Challenges with private sector including financial incentives, lack of cost subsidy and poor results notification |
| Clouse 2012  South Africa | POC Xpert at primary care clinics | Presumptive TB patients at NGO operated clinic, informal settlement area | Operational research | Facility | Implementation in primary care is feasible with financial, logistical and operational support | High personnel needs, insufficient equipment, clinic congestion |
| Cowan 2015  Mozambique | Xpert at district and urban hospitals with rural specimens transferred for testing, transportation network for smear negative specimens | Presumptive TB patients at urban and rural hospitals | Mixed methods | Provider  Facility  Data | Staff education, use of native language for Xpert operation, SMS transmission of results | System capacity, maintenance of equipment, loss to follow-up, language barriers |
| Hanrahan 2016  Uganda | Analysis of on-site versus centralized Xpert at 18 facilities in Uganda (regional and district hospitals and sub-district health centers) | Presumed TB: PLHIV and smear negative, children, HWs, or contacts of MDR-TB cases | Operational research | Health systems  Provider | Supportive infrastructure  and phone reminders | Technical difficulties, test stockouts and empiric TB treatment |
| Lessells 2017  South Africa | POC Xpert placed in clinic versus in centralized sub-district lab | Adults with possible PTB/DR-TB, rural | Cluster RCT | Facility  Patient | POC placement increased access to Xpert | LTFU and delays with centralized testing, restricted operating hours even with POC testing |
| Ndlovu 2018  Zimbabwe | Integrated HIV VL, EID and MTB/RIF testing in new GeneXpert platform | Decentralized in district and subdistrict | Operational research:  Prospective field feasibility study | Health systems  Facility  Data | Xpert is easy to learn to use and prior IT training helps, internal/external quality control, sample transport, remote connectivity and digital results reporting | Physical environment, lack of collaboration between national TB and HIV programs, costs and need to prioritize samples in decentralized testing |
| Ngwira 2019  Malawi | POC Xpert on single expectorated sputum | Symptom screen-positive patients at time of HIV diagnosis, rural primary health clinic | Cluster RCT | Facility | Xpert is easy for non-laboratory trained staff to use | Power supply, maintenance and supplies, training personnel |
| Schumacher 2015  India | POC Xpert in clinic by non-laboratory personnel, expedited sample transport, rapid reporting and follow up | Presumptive TB, urban outpatient clinic | Operational research | Facility  Data | Streamlining laboratory protocols, use of mHealth technology | Limited Xpert throughput,  delayed results retrieval by clinicians |
| Stime 2018  South Africa | Integrated POC testing using Xpert along with STI and HIV testing on site at a public clinic | Patients being screened for TB | Mixed methods | Facility | POC placement improves access | Poor clinic workflow leading to long wait times and time to treatment |
| Van den Handel 2015  South Africa | Decentralized Xpert placement | Laboratory staff, rural | Operational research:  Non-randomized interventional study | Health systems  Facility | Decentralized placement improved access and same day treatment for patients | HWs restricted use of Xpert at facility level |
| Zishiri 2015  South Africa | Xpert used following symptom screening in prisoners | Prison inmates, correctional facilities | Mixed methods | Health systems  Facility | Access for high-risk, underserved population | Limited capacity of 4-module Xpert |
| **Use of mWRDs** |  |  |  |  |  |  |
| Abdurrahman 2015  Nigeria | Pooled sputum Xpert testing | Presumptive TB patients in hospitals and community | Operational research:  Cross-sectional | Health systems | Use of pooled testing | High cost of Xpert, low community prevalence |
| Colvin 2015  South Africa | Implementation of Xpert in South Africa | National policy analysis | Operational research:  Policy transfer analysis framework | Health systems | Analysis of global to local policy transfer to examine uptake and delivery of Xpert (along with line probe assays) in South Africa | Lack of health system integration, technical mWRD challenges, stockouts, training needs, unintended consequences of donor support |
| Creswell 2014  Bangladesh  Cambodia  DR Congo  Kenya  Malawi  Moldova  Mozambique  Nepal  Pakistan | Varied- operational reports focused on use of Xpert platforms in different settings | Varied- included public and private facilities, PLHIV, prisons | Mixed methods | Health systems  Facility | Staff satisfaction, needs assessment, maintenance support, screening with CXR prior to using Xpert | Machine utilization, testing algorithms, time to diagnosis, procurement, training, infrastructure, test failure |
| Dabas 2019  India | Market based approach to develop low-cost high-volume approach to promote Xpert in Indian private sector | Private providers evaluating people with potential TB, private sector | Operational research | Health systems  Provider | Competitive tendering by suppliers  Digital knowledge dissemination  Expansion to include HIV and HCV | Xpert price  Private provider perspectives |
| Deo 2020  India | Field sales force demand generation model | Private providers evaluating people with potential TB, private sector | Operational research | Health systems  Provider | Expansion to include HIV and HCV, ROI may be more attractive for 1 or 2 module Xpert | Distorted financial incentives for private providers and financial barriers for patients, tendency to prescribe empiric TB treatment |
| Deo 2021  India | Private provider engagement program to increase Xpert uptake | Private providers evaluating people with potential TB, private sector | Operational research | Health systems  Provider | Addressing provider level behaviors, Xpert free of charge for patients | High variability in provider behaviors between cities |
| Durovni 2014  Brazil | Replacing two sample smear with one sample Xpert | Presumptive TB,  Urban, primary care laboratories | Stepped wedge cluster RCT | Health systems  Facility | Training personnel  Uninterrupted power supply  Laboratory information system | Lack of unique identifiers  Laboratory capacity  Technical barriers to Xpert testing |
| Gidado 2018  Nigeria | Scale up of Xpert at the national level using GxAlert | People being tested for TB, urban and rural | Operational research | Health systems  Facility  Data | Quality supervision and mentoring | Technical errors – both related to human processing and module failures |
| Manabe 2015  Uganda | Bundled or multicomponent lab diagnostics and on-site training, Xpert only used in smear-negative PLHIV | Presumptive TB, rural healthcare facilities | Quasi-experimental intervention study | Health systems  Facility | Training, improved infrastructure | No financial incentive for staff,  weak health care systems |
| McDowell 2018  India | Free of cost Xpert for paediatric TB diagnosis using hub and spoke model available to both public and private providers identified through mapping | Paediatricians | Qualitative | Health systems  Provider | Value of Xpert to identify drug resistance and reduce time to diagnosis | High threshold to perform Xpert  Limitations of test accuracy in children  Sample collection issues |
| Paudel 2021  Nepal | Active case finding, FAST. Xpert used for those with positive cough screen | Symptom-screen positive patients at Nepal hospital | Qualitative- key informant interviews | Facility | - | Limited capacity, cartridge supply, maintenance |
| Pho 2015  Uganda | Modelling Xpert for smear negative vs replacing smear microscopy altogether | Clinics, labs, hospitals in urban and rural settings | Operational research: Theoretical model with epidemiologic and operational data | Health systems | Xpert placement may be most effective in high TB and HIV prevalence areas and in sites with poor sputum microscopy performance; important to use program level data to inform Xpert placement | - |
| Shete 2017  Uganda | SIMPLE-TB: single spot specimen for smear, if two negative smears sample transported to Xpert testing sites | Patients and staff and peripheral health clinics | Operational research: Single arm interventional pilot study | Facility | Streamlined process with single sample for patients, use of fluorescence microscopy,  daily sputum transport | Prolonged diagnostic process requiring multiple days, high cost and infrastructure requirements for Xpert |
| Shibu 2020  India | PPIA to develop referral network, provision of Xpert, support services, monitoring throughout cascade | Private providers and laboratories | Operational research | Health systems | Private sector engagement  Financial strategies | Patients initially had to pay for Xpert  Dominant private sector |
| **Diagnosis using mWRDs and results delivery** |  |  |  |  |  |  |
| Babirye 2019  Uganda | Use of automated SMS to deliver Xpert results using GxAlert software | Presumptive TB undergoing Xpert at peripheral health centres | Operational research:  Single arm interventional pilot study | Data | Many people have cell phones, patients who received results could relay these, similar by gender/age | High PTLFU and many did not have phone or wish to share number, lack of infrastructure to receive results |
| Cowan 2016  Mozambique | Pilot testing GxAlert (remote monitoring to disseminate real-time Xpert results) | 5 public health centres | Operational research | Data | Strong/established system  and simple to install. User friendly interface, uses cloud per server. Real time performance feedback | Lack of electronic records, including for storage of Xpert result  Connectivity, software |
| **Notification and treatment initiation** |  |  |  |  |  |  |
| Alagna 2020  Burkina Faso | Xpert network strengthening (ASAP-GxNet) | Urban settings at national level | Operational research:  Pre/post evaluation | Health systems | Customised training with longitudinal support, external mentorship with lab strengthening expertise,  dedicated funding | - |
| **Multiple barriers addressed** |  |  |  |  |  |  |
| Cattamanchi 2020  Uganda | Development of multicomponent SIMPLE-TB strategy | Presumptive TB patients at peripheral health centres | Mixed methods, use of PRECEDE framework | Health systems  Facility  Data | Streamlining the care cascade with daily specimen transport, SMS communication, performance feedback | Delays in Xpert referral networks, time/distance/cost to access care  HW knowledge and training gaps,  staff turnover, specimen transport |
| Cattamanchi 2021  Nalugwa 2021  Nalugwa 2020  Reza 2021  Uganda | Multicomponent strategy with decentralized/onsite Xpert, process design and performance feedback | Presumptive TB patients presenting to peripheral care sites | Cluster RCT, ultra-pragmatic design, Mixed methods | Facility | Leadership and staff culture and timely results delivery facilitated mWRD implementation | Technical skills, equipment storage, unstable power, safe disposal,  sputum collection, storage, delayed notification |
| Jeyashree 2020  India | Evaluation of Truenat in POC TB unit settings | Presumptive TB, rural | Operational research | Health systems  Facility | Manpower and external quality assurance can help with Truenat implementation | Staffing challenges, variation in results |
| Khushvakhtov 2021  Tajikistan | Scale up of Xpert with GxAlert and Open MRS system | TB centers and TB diagnostic laboratories that used Open MRS, GeneXpert and GxAlert, patients diagnosed using Xpert | Operational research-longitudinal analysis including historical cohorts | Health systems  Data | Roll out of country wide open MRS medical information system | Weak linkage to care  Data and communications breakdown |
| Lisboa 2020  Mozambique | Use of hospital auxiliary workers and 24 hour Xpert access | Presumptive TB, two hospital sites | Quasi-experimental intervention study | Health systems  Facility | Use of auxiliary workers to support clinicians and expansion of testing hours | Lack of dedicated logistical staff, nurse overwhelm  Limited TB laboratory operational hours |
| Nathavitharana 2017  Bangladesh | As part of FAST algorithm for active case finding | Tertiary hospitals in urban setting | Operational research:  Cross sectional | Health systems  Facility | Rewarding staff who need to task-shift to operate Xpert, use of 16 cartridge machine | Staff shortages, diagnostic/module failure, supply-chain issues, reliance on external funding, sample transport, lack of on-site testing, lack of experience using Xpert |
| Raizada 2018  India | Hub and spoke model of Xpert placement, provider engagement, use of Xpert on different specimen types for pediatric patients | Symptomatic paediatric patients; urban clinics, labs, hospitals | Operational research: Pre-post intervention, mixed methods | Health systems  Facility  Patient | A multi-component strategy with hub and spoke model, strengthening sample transport, staffing, provider outreach and education, and electronic results communication facilitated implementation | Provider delay in prescribing Xpert, private sector engagement, difficulty in collecting non-sputum samples |
| Theron 2014  South Africa, Zimbabwe, Zambia, and Tanzania | Decentralized placement- Xpert done by nurses vs smear | Symptomatic patients presenting to urban primary care centers | RCT | Facility | POC Xpert improved access for patients, could be performed by nurses | Empiric treatment |
| Umubyeyi 2016  Multi-country | Technical assistance for Xpert scale up | TB programme managers, Urban and rural | Operational research | Health systems | Technical support  HSS  Coordinated donor support | Laboratory infrastructure  Empiric treatment |
| Vatsyayan 2022  India | DOST model sought to effectively link and refer private DR-TB patients to programmatic DR-TB centers and provide treatment adherence support | People with DR-TB, urban | Operational research:  Cross-sectional | Health systems  Provider | Longitudinal engagement of private sector providers e.g. through use of multi-media approach for education and outreach, real-time patient monitoring and use of mHealth and social media. | Gaps and delays in DR-TB care |
| Yuen 2021  Peru | Xpert used as part of mobile ACF strategy | Community members, urban | Operational research:  Pre-post intervention study | Patient  Health systems | Use of mass community screening to test high-risk population | Need for health worker training regarding test interpretation to reduce treatment initiation delays |
| Zawedde-Muyanja 2022  Uganda | Multicomponent- education to improve HW knowledge, redesigned laboratory forms and workflow | Staff: HWs, laboratory staff, managers, hospital and primary care facilities | Mixed methods | Health systems  Provider | Use of other POC tests like LAM  Batched delivery of specimens at end of day  On demand specimen analysis | Patient lack of transport fares to return  Reduced laboratory staffing  TB clinic closure after hours |
| Systematic reviews on mWRD implementation | | | | | | |
| Brown 2021 | Analysis of barriers and enablers | Data from different populations and settings | Systematic review | Multiple | Examination of multi-level barriers | Commonality of implementation barriers across geographic settings where Xpert has been implemented and need for implementation frameworks for reporting |
| Engel 2022 | Analysis of user perspectives | Data from different populations and settings | Systematic review | Multiple | Qualitative synthesis of user perspectives related to mWRDs | Lack of infrastructure and human resources undermine added value of mWRDs for patient and health system users |
