# Supplementary material for: Implementation strategies to increase the uptake and impact of molecular WHO-recommended rapid diagnostic tests: evidence from a mixed-methods systematic review
Source: BMJ Glob Health. 2025 Sep 17;10(9):e018700. doi: 10.1136/bmjgh-2024-018700 (PMC12458786; doi:10.1136/bmjgh-2024-018700)
Supplement: online supplemental file 4 [file bmjgh-10-9-s004.docx]

| **Study** | **mWRD implementation strategy** | **Country** | **Population, setting** | **Design** | **Barriers** | **Enablers** | **User outcomes** | **System outcomes** | **Implementation outcomes** |
| --- | --- | --- | --- | --- | --- | --- | --- | --- | --- |
| Abdurrahman 2015 | Pooled sputum Xpert testing | Nigeria | Presumptive TB, attending hospitals and identified in community | Operational research: Cross-sectional | High cost of Xpert  Low community prevalence | Pooled testing | Required CHWs to identify people at risk | Could decrease ACF costs particularly if used for community screening | Feasible  Accurate compared to individual testing, with  reduction in time and costs |
| Agizew 2017 | Centralized lab vs. POC Xpert | Botswana | People identified for TB testing ?at peripheral clinics | Stepped wedge trial | Lab staff rotation and turnover  Training  Perceptions | Refresher training  Strengthening infrastructure  Supervisor support | Workload | High rate of invalid tests decreased over time, errors persisted | Feasible to place Xpert nearer to patient |
| Alagna 2020 | Xpert network strengthening (ASAP-GxNet) | Burkina Faso | Urban settings at national level | Operational research: Pre/post evaluation | External mentorship with lab strengthening expertise  Dedicated funding | Customised training with longitudinal support | N/A | Electronic systems, diagnostic optimization  Staffing | Adoption  Penetration  Sustainability |
| Albert 2020 | Diagnostic network optimization | Lesotho | Country wide | Operational research | Underutilization of Xpert  Sample transportation  Linking people with symptoms and tests  Multiple partners/donors | Relocating Xpert  Sample transport support  Integrated TB HIV care  POC platforms like Omni | N/A | Relocating Xpert platforms had similar impact to procuring new ones | Feasible to more closely match demand by optimizing transportation and sourcing |
| Awan 2018 | Multicomponent reverse PPM targeting public hospitals and PMDT sites and SBM targeting private sector | Pakistan | Presumptive TB, public and private sector facilities in urban city | Operational research: Cross-sectional intervention | Costs for equipment maintenance and support  Supply chains  Poor public-private integration | Use of community-based screeners  Social business model using medical detailing  Free treatment | Not measured | Increased DR-TB notification  Need for data systems, human resource training | Feasible and acceptable in public and private sector settings |
| Babirye 2019 | Use of automated SMS to deliver Xpert results using GxAlert software | Uganda | Presumptive TB undergoing Xpert at peripheral health centres | Operational research: Single arm interventional pilot study | High PTLFU  33% did not have phone or wish to share number  Lack of infrastructure to receive results | Many people have cell phones | Patients who received results could relay these, similar by gender/age  May not be as favourable as call | Health centres could receive results, may support digitalization of TB records | Feasible to deliver results by automated SMS: health centers confirmed receipt in 95%, but only 49% of patients  High fidelity |
| Banu 2020 | Multicomponent- 3 TB screening centres with free Xpert to which patients could be referred by public and private providers (latter engaged through social enterprise model) | Bangladesh | Presumptive TB, urban | Operational research: Cross-sectional intervention | Lack of treatment and notifications in the private sector  Private sector patients not eligible for subsidized Xpert  Commercial incentives within private healthcare markets | Subsidized digital CXR  Free Xpert  Radiography revenues supported screening centre costs  Patients could choose private vs public | Patient-centred re: choice of public vs private  Private providers treating more people than are sent for testing | Increased case detection and bacteriological confirmation | Demonstrates feasibility and impact of linking private and public sector including increasing DR-TB diagnoses  Highlights potential for dCXR as a triage tool  SEM recovered 37% of operational costs |
| Brown 2021 | Analysis of barriers and enablers | Multi-country |  | Systematic review |  |  |  |  |  |
| Cattamanchi 2020 | Development of multicomponent SIMPLE-TB strategy | Uganda | Presumptive TB, peripheral health centres | Mixed methods, use of PRECEDE framework | Delays in Xpert referral networks  Time/distance/cost to acess care  HW knowledge and training gaps  Staff turnover  Specimen transport | Same day diagnosis and treatment  Daily specimen transport  SMS communication  Performance feedback | Reduced patient time and costs re: diagnosis | QI feedback, infrastructure changes to reduce TAT and improve linkage | Demonstrated >99% referred and completed sputum Xpert testing and 86% treated within 14d i.e. feasibility and effectiveness |
| Cattamanchi 2021  Nalugwa 2021  Nalugwa  Reza 2021 | Multicomponent strategy with decentralized/onsite Xpert, process design and performance feedback | Uganda | Presumptive TB, presenting to peripheral care sites | Cluster RCT, ultra pragmatic design, Mixed methods | Technical skills, equipment storage, unstable power, safe disposal  Sputum collection, storage  Delayed notification | Technical simplicity of mWRD  Reduced time to results  Health centre director leadership  Staff culture | Improved patient & provider relationship through timely results delivery  Improved HW self-efficacy | Improving access through same day near-patient testing  Training  QI feedback  Service integration | Effective based on 14 day Rx initiation metric  Other key cascade indicators such as time to diagnosis also improved |
| Clouse 2012 | POC Xpert at primary care clinics | South Africa | Presumptive TB, NGO operated clinic, informal settlement area | Operational research | Human resources, time, personnel, high instrument requirements  Clinic congestion | Limited need for specialized skills  Clinic staff enthusiastic about same day result | High personnel needs | Considerable human resources | POC Xpert feasible but required financial, operational, and logistical support. |
| Colvin 2015 | Implementation of Xpert in South Africa | South Africa | National policy analysis | Operational research: Policy transfer analysis framework using | Poor coordination & commmunication  Lack of health system integration  Technical mWRD challenges, stockouts, training needs  Unintended consequences of donor support | Tugela Ferry XDR outbreak catalyzed need for mWRD | N/A | Equity issue re: placement of mWRD in better functioning districts  Service integration  Messaging  Media | Poorly coordinated implementation  Pace of roll out left little time to assess operation and integration  Concern re: data showing limited impact on case finding, morbidity and mortality |
| Cowan 2015 | Xpert at district and urban hospitals with rural specimens transferred for testing, transportation network for smear negative specimens | Mozambique | Presumptive TB | Mixed methods | System capacity aid increased demand on diagnostic services  Inconsistent |  |  |  | Increased number and proportion of bacteriologically confirmed TB |
| Cowan 2016 | Pilot testing GxAlert (remote monitoring to disseminate real-time Xpert results) | Mozambique | 5 public health centres | Operational research | Lack of electronic register for cough, laboratory results or patient records  Xpert data extracted manually preventing management of Xpert inventory or notification particularly for DR-TB  Connectivity, software | Strong/established HIV system  Simpler one -click installation package | User friendly interface, uses cloud per server  Real time performance feedback | New version of Xpert | Feasible  Successful but complex |
| Creswell 2014 | Varied- operational reports focused on use of Xpert platforms in different settings | Multicountry:  Bangladesh  Cambodia  DR Congo  Kenya  Malawi  Moldova  Mozambique  Nepal  Pakistan | Varied- included public and private facilities, PLHIV, prisons | Mixed methods | Machine utilization  Testing algorithms  Time to diagnosis  Procurement  Training  Infrastructure  Test failure |  |  |  |  |
| Dabas 2019 | Market based approach to develop low-cost high volume approach to promote Xpert in Indian private sector | India | Private providers evaluating people with potential TB, private sector | Operational research | Xpert price  Private provider perspectives | Competitive tendering by suppliers  Digital knowledge dissemination  Expansion to include HIV and HCV | Enables patients to choose private sector care  Enables providers to retain patients in their care | Need to integrate private and public services  Use of digital technology for engagement and linkage | Market-based intervention was feasible and effective Challenges estimated impact of market-level intervention |
| Deo 2020 | Field sales force demand generation model | India | Private providers evaluating people with potential TB, private sector | Operational research | Use of empiric treatment  Financial incentives for non-recommended tests  Need for external donor funding  Patient out-of-pocket costs | Expansion to include HIV and HCV  ROI may be more attractive for 1 or 2 module Xpert | Patient care and access  Provider education and support | Service integration  Linkage between private and public sector | Acceptability  Adoption  Feasibility  Effectiveness |
| Deo 2021 | Private provider engagement program to increase Xpert uptake | India | Private providers evaluating people with potential TB, private sector | Operational research | High variability in provider behaviors between cities | Xpert free of charge for patients | Addressing provider level behaviors | Consideration of high volume providers | Effectiveness- uptake increased however upfront utilization of Xpert did not increase and rates of treatment following negative results did not decrease |
| Durovni 2014 | Replacing two sample smear with one sample Xpert | Brazil | Presumptive TB,  Urban, primary care laboratories | Stepped wedge cluster RCT | Lack of unique identifiers  Laboratory capacity  Technical barriers to Xpert testing | Training personnel  Uninterrupted power supply  Laboratory information system | Laboratory provider training and skills | Improving training and staffing Improving files and records | Feasible to replace smear  Effective- although notification rate did not decrease, time to treatment decreased although was still >1 week. |
| Engel 2022 | Analysis of user perspectives | Multi-country |  | Systematic review |  |  |  |  |  |
| Gidado 2018 | Scale up of Xpert at the national level using GxAlert | Nigeria | People being tested for TB, urban and rural | Operational research | Technical errors – both related to human processing and module failures | Quality supervision and mentoring | Ensuring access where patients present | Various infrastructural issues including power across health facility levels | Overall underutilization of Xpert  Improved function rather than new machines  Rate of unsuccessful tests is an important indicator |
| Hanrahan 2016 | Comparison of programmatic data from facilities where Xpert was implemented on-site vs centralized | Uganda | Presumed TB: PLHIV and smear negative, children, HWs, or contacts of MDR-TB cases | Operational research | Technical difficulties  Test stockouts  Empiric treatment | Supportive infrastructure  Phone reminders | N/A laboratory record data | Machine procurement and training alone is insufficient | Low Xpert utilization  No difference in proportion of presumptive TB started on treatment or time to treatment initiation |
| Jeyashree 2020 | Evaluation of Truenat in POC TB unit settings | India | Presumptive TB, state with 70% rural residents | Operational research |  |  |  |  |  |
| Khushvakhtov 2021 | Scale up of Xpert with GxAlert and Open MRS system | Tajikistan | TB centers and TB diagnostic laboratories that used Open MRS, GeneXpert and GxAlert, patients diagnosed using Xpert | Operational research-longitudinal analysis including historical cohorts | Weak linkage to care  Data and communications breakdown | Roll out of country wide open MRS medical information system | N/A |  | Reduction in time between diagnosis and treatment for those who previously had >2 weeks delay  Data quality only improved slightly  Challenges linking Xpert to GxAlert |
| Lessells 2017 | POC Xpert placed in clinic versus in centralized sub-district lab | South Africa | Adults with possible PTB/DR-TB, rural | Cluster RCT | LTFU and delays with centralized testing  Restricted operating hours even with POC testing |  | Not captured, ease of access with POC testing for patients | Increased Xpert costs, need more instruments and staff with decreased operational efficiency at the clinic level | Primarily focused on effectiveness – shorter time to treatment initiation but failure to achieve same day treatment due to patients not being able to wait and restricted operating hours |
| Lisboa 2020 | Use of hospital auxillary workers and 24 hour Xpert access | Mozambique | Presumptive TB, two hospital sites | Quasi-experimental intervention study | Lack of dedicated logistical staff, nurse overwhelm  Limited TB laboratory operational hours | Use of auxillary workers |  | Streamlined processes including immediate collection of sputum | Good fidelity to intervention and strategy  Earlier time to diagnosis, reduced time to treatment and lower mortality in intervention hospital |
| Manabe 2015 | Bundled or multicomponent lab diagnostics and on-site training, Xpert only used in smear-negative PLHIV | Uganda | Presumptive TB, rural healthcare facilities | Quasi-experimental intervention study | Limited test sensitivity  Weak health care systems | Training  Improved infrastructure | No pay for performance or additional salary support for staff | Equipped and improved healthcare facility capacity | Increased proportion of people undergoing sputum based testing and microbiologically conformed TB |
| McDowell 2018 | Free of cost Xpert for paediatric TB diagnosis using hub and spoke model available to both public and private providers identified through mapping | India | Paediatricians | Qualitative | Limitations of test accuracy in children  Sample collection issues | Value of Xpert to identify drug resistance and reduce time to diagnosis | Improved access for patients  Value of mWRDs for clinicians although often high threshold to perform test | Laboratory infrastructure strenghtening  Staffing including training | Xpert adopted by clinicians  Importance of trust in infrastructure |
| Medina-Marino 2021 | Home-based testing using modified portable Xpert instrument (GX1) | South Africa | Household TB contacts | Qualitative (nested within RCT) | Low referral rates for HHCs  mWRDs always facility-based  Time consuming due to technical challenges  Issues re: disclosure at home | Mobile mWRDs enable home testing | Access for patients  Person-centred care | Outreach | Home-based testing was acceptable & feasible |
| Nathavitharana 2017 | As part of FAST algorithm for active case finding | Bangladesh | Tertiary hospitals in urban setting | Operational research: Cross sectional | Staff shortages, diagnostic failure, supply-chain issues, reliance on external funding, sample transport, lack of on-site testing, lack of experience using Xpert, frequent module failure | Rewarding staff who need to task-shift to operate Xpert, 16 cartridge machine |  | Increased detection of patients with unsuspected and MDR TB as part of FAST. | Xpert feasible as part of FAST algorithm for active case finding |
| Ndlovu 2018 | Integrated HIV VL, EID and MTB/RIF testing in new GeneXpert platform | Zimbabwe | Decentralized in district and subdistrict | Operational research: Prospective field feasibility study | Physical environment, lack of collaboration between national TB and HIV programs, costs and need to prioritize samples in decentralized tesing | Minimal training needed to use Xpert and prior IT training helps, internal/external quality control, sample transport, remote connectivity and digital results reporting | High laboratory staff satisfaction levels in inteviews | Improved TB and HIV care integration | Integrated multi-disease POC testing is feasible. Shorter TAT compared to centralized testing |
| Ngwira 2019 | POC Xpert on single expectorated sputum | Malawi | Symptom screen-positive patients at time of HIV diagnosis, rural primary health clinic | Cluster RCT | Power supply, training personnel, maintenance and supplies | Can be performed by nurses |  | Considerable infrastructure changes required | Xpert at time of HIV Dx increased diagnosis of patients with TB compared to sputum and decreased mortality in advanced AIDS patients |
| Page-Shipp 2014 | POC Xpert at public event | South Africa | Symptomatic patients from urban gold mining community, mobile testing vehicle | Operational research: Cross sectional | Web connectivity needed, back up generators needed | Back-up electrical supply |  |  | Mobile testing at public events is feasible but low yield |
| Paudel 2021 | Active case finding, FAST. Xpert used for those with positive cough screen | Nepal | Symptom-screen positive patients at Nepal hospital | Qualitative- key informant interviews | Xpert machine capacity, inadequate cartridge supply, lack of maintenance technicians |  |  |  | FAST may be feasible, but various barriers must be addressed |
| Pho 2015 | Modelling Xpert for smear negative vs replacing smear microscopy altogether | Data from Uganda | Clinics, labs, hospitals in urban and rural settings | Operational research: Theoretical model with epidemiologic and operational data |  |  |  | Placement strategies that prioritized sites with higher TB prevalence  maximized CDR. Important to use program level data to inform Xpert placement | Xpert placement may be most effective in high TB and HIV prevalence areas and in sites with poor sputum microscopy performance |
| Raizada 2018 | Hub and spoke model of Xpert placement, provider engagement, use of Xpert on different specimen types for pediatric patients | India | Symptomatic paediatric patients; urban clinics, labs, hospitals | Operational research: Pre-post intervention, mixed methods | Provider delay in prescribing Xpert (“test of last resort”), private sector engagement, difficulty in collecting non-sputum samples | Increased cost effectiveness of hub and spoke (sending samples to one lab with Xpert), good performance on non-sputum samples, improved sample transport, staffing, provider outreach and education, ease of use of Xpert, electronic results communication |  |  | Hub and spoke model is a feasible strategy, when combined with rapid sample transport and provider engagement. May be useful in diagnosis of children as it has good performance on non-respiratory samples. |
| Schumacher 2015 | POC Xpert in clinic done by non-laboratory personnel, expedited sample transport, rapid reporting and follow up | India | Presumptive TB, outpatient clinic, urban | Operational research | Limited Xpert throughput  Delayed results retrieval by clinicians  Diagnostic delays due to work hours | Streamlining laboratory protocols  Use of mHealth technology | Improved near-patient access | Innovated clinic processes, required dedicated staff and revised workflow | Benefit of Xpert as POC test with reduction in time to diagnosis, including same day diagnosis and reduced time to treatment |
| Shete 2017 | SIMPLE-TB: single spot specimen for smear, if two negative smears sample transported to Xpert testing sites | Uganda | Patients and staff and peripheral health clinics | Operational research: Single arm interventional pilot study | Suboptimal sensitivity of smear  Prolonged diagnostic process requiring multiple days  High cost and infrastructure requirements for Xpert | Restructuring clinic procedures with single sample  Use of fluorescence microscopy  Daily sputum transport | Streamlined process with single sample for patients | Staff training  Process redesign | Feasible to deliver strategy with fidelity  High incremental diagnostic yield  Decreased LTFU but 15% of patients with positive Xpert results were not initiated on treatment |
| Shibu 2020 | PPIA to develop referral network, provision of Xpert, support services, monitoring throughout cascade | India | Private providers and laboratories | Operational research | Patients initially had to pay for Xpert  Dominant private sector with increased costs and concerns re: quality of care | Private sector engagement  Financial strategies | Patient-centred care | Diagnostic strengthening  Service integration between public and private | Feasible to effectively engage private providers and increase TB case notification rate |
| Stime 2018 | Integrated POC testing using Xpert along with STI and HIV testing on site at a public clinic | South Africa | Patients being screened for TB | Mixed methods | Long waiting times  POC testing did not mean same day treatment  Time inefficiency compared to syndromic treatment | POC placement improves TAT | Improved access | Laboratory infrastructure, staff shortages and training  Needs clinic workflow re-design to reduce bottlenecks | Few people tested and initiated on same-day treatment |
| Theron 2014 | Decentralized placement- Xpert done by nurses vs smear | South Africa, Zimbabwe, Zambia, and Tanzania | Symptomatic patients presenting to primary care centres, urban | RCT | Delayed diagnosis  Empiric treatment | POC placement of Xpert to enable same-day diagnosis and treatment | Improved access for patients  Expanded scope of work for nurses | Questions regarding personnel who can/should do mWRD in peripheral settings  Variation in practices across study sites | Feasible to train non-laboratory personnel.  Increased rates of same-day treatment, treatment initiation, and decreased time to treatment, but did not change morbidity, presumed due to high rates of empirical treatment in smear negative patients. |
| Umubyeyi 2016 | Technical assistance for Xpert scale up | Multi-country | TB programme managers, Urban and rural | Operational research | Laboratory infrastructure  Empiric treatment | Technical support  HSS  Coordinated donor support | - | Need for coordination between partners | Increased number with bacteriologic confirmation otherwise mixed effectiveness results |
| VandenHandel 2015 | Decentralized Xpert placement | South Africa | Laboratory staff, rural | Operational research: Non-randomized interventional study | HWs restricted use of Xpert at facility level | Decentralized placement | Improved access and same day treatment for patients | Concerns related to cost at facility level | Increased number with bacteriologic confirmation |
| Vatsyayan 2022 | DOST model sought to effectively link and refer private DR-TB patients to programmatic DR-TB centers and provide treatment adherence support | India | People with DR-TB, urban | Operational research: Cross-sectional | Gaps and delays in DR-TB care | Longitudinal provider engagement  Use of multi-media approach for education and outreach  Real-time patient monitoring  Use of mHealth and social media | Reduction in multiple visits made by patients for evaluation | Need for one-stop public health facility  mHealth can improve connectivity and linkage | Improved testing rates and linkage between private and public sector |
| Yuen 2021 | Xpert used as part of mobile ACF strategy | Peru | Community members, urban | Operational research: Pre-post intervention study | Lack of education about Xpert  Lack of patient social protection | HW training | Improved access for patients | Despite diagnosis using Xpert, patients then referred for re-evaluation by pulmonologists prior to treatment | Xpert as part of mobile screening approach is feasible, acceptable, and could be adopted with fidelity |
| Zawedde-Muyanja 2022 | Multicomponent- education to improve HW knowledge, redesigned laboratory forms and workflow | Uganda | Staff: HWs, laboratory staff, managers, hospital and primary care faciltiies | Mixed methods | Patient lack of transport fares to return  Reduced laboratory staffing  TB clinic closure after hours | Use of other POC tests like LAM  Batched delivery of specimens at end of day  On demand specimen analysis |  | Improved notifications, files and records, staffing including training | Reduced test TAT and time to treatment initiation within 2 weeks |
| Zishiri 2015 | Xpert used following symptom screening in prisoners | South Africa | Prison inmates, correctional facilities | Mixed methods | Limited capacity of 4-module Xpert | - | Access for high-risk population | Improved efficiency | Xpert testing was feasible and performed with fidelity when adopted in correctional facilities  Equity |
