# Supplementary material for: Implementation strategies to increase the uptake and impact of molecular WHO-recommended rapid diagnostic tests: evidence from a mixed-methods systematic review
Source: BMJ Glob Health. 2025 Sep 17;10(9):e018700. doi: 10.1136/bmjgh-2024-018700 (PMC12458786; doi:10.1136/bmjgh-2024-018700)
Supplement: online supplemental file 5 [file bmjgh-10-9-s005.docx]

**Table S3. Characteristics of studies of mWRD implementation, medium thickness studies**

| **Study** | **Country** | **Design, population** | **Setting** | **mWRD** | **Barriers** | **Enablers** | **Comments** |
| --- | --- | --- | --- | --- | --- | --- | --- |
| Abdurrahman 2014 | Nigeria | Laboratory-based | Installation of Xpert at 5 sites | Xpert | Installation involves costs, such as extra equipment to secure uninterrupted electrical supply and additional laboratory space | Anticipate hidden costs | TB-REACH project |
| Abebaw 2022 | Ethiopia | Laboratory-based, N = 34 facilities | Randomly selected Xpert testing health facilities | Xpert | ~ 50% facilities did not monitor delay of samples after collection; feedback from providers and results reporting | Increase onsite supervision; improve sample transport proficiency testing and communication with providers | QA study; not an implementation study |
| Agonafir 2018 | Ethiopia | Healthcare providers, N = 209 | TB clinics in Addis Ababa | Xpert | Insufficient knowledge about position of Xpert in the diagnostic pathway | Training |  |
| Alemu 2019 | Ethiopia | Laboratory-based, N = 2515 samples, 8 testers | National TB Reference Laboratory | Xpert | 9.6% unsuccessful results owing to results error; most resolved with repeat testing; TAT was < target | Monitor laboratory performance indicators; training | QA study; not an implementation study |
| Amicosante 2017 | Multi-country | N = 723 respondents from 114 countries | Large structured survey conducted in 2016 | Rapid molecular tests in general | Education, cost | Training and education of end users | 86% respondents stated they would accept novel rapid TB tests, including molecular tests, if available; acceptance significantly associated with higher education level although not with having decision-making role or years of experience in TB |
| Ardizzoni 2015 | 18 countries | People presumed to have MDR-TB or HIV-associated TB, N = 52,863 samples; 38 Xpert machines in 33 projects | Settings: district and sub-district labs with range of TB, MDR-TB, and HIV prevalence; (21/33), 5 regional, 6 peripheral, 1 penal facility | Xpert | Operational and logistical hurdles: highlighted installation of air conditioning (54%) and generator (39%) | Infrastructure renovation, basic computer training, regular instrument troubleshooting and maintenance, substantial, continuous support | MSF project; reported key lessons learned during 2011-12 in implementation of Xpert under routine conditions; note 23 sites used Xpert as initial test for all people with presumptive TB |
| Auld 2014 | Cambodia | PLHIV, N = 497 participants | Mainly TB and HIV clinics; specimens were transported to referral hospital for testing | Xpert | Training delays and challenges with specimen referral may have contributed to variable Xpert uptake and TAT, particularly for sites without onsite Xpert testing | Programmatic support for specimen referral and results reporting |  |
| Banamu 2019 | Papua New Guinea | Retrospective pre-post study of patients with RR-TB, N = 172 participants | Referral hospital | Xpert | Pre-treatment attrition and time to treatment initiation | GxAlert | Study suggests impact of an electronic results notification tool for Xpert on time to treatment initiation and patient outcomes; also found significant decrease in pre-treatment attrition following introduction of GxAlert |
| Beste 2018 | Mozambique | Retrospective clinical review of patients with RR-TB, N = 32,182 Xpert tests | 5 Xpert sites, 8 facilities that referred samples from people with presumptive TB and MDR-TB for Xpert testing to these sites | Xpert | Highlights ongoing challenges in linkage to care, as only 52.6% of patients with RR-TB diagnosed by Xpert were started on appropriate MDR-TB treatment | Hypothesize that SMS and GxAlert could be more effective if could fully leverage and integrate community health workers and socioeconomic interventions | GxAlert did not affect treatment initiation rates |
| Boehme 2011 | Multi-country | Prospective study, N = 6648 participants, people with presumed TB and risk for MDR-TB | District and subdistrict level health facilities | Xpert | Xpert maintenance requirements | Decentralized Xpert testing is feasible; operator training | Seminal study on Xpert implementation; findings support laboratory managers’ perception that performance of Xpert might be less dependent on user skills, motivation, or workload than microscopy; operators without previous molecular biology experience or computer skills passed proficiency testing after 1–3 days of training |
| Cassim 2021 | South Africa | N = 4866 tests | 258 healthcare facilities were visited | Xpert | Staff, reagents, laboratory equipment, and vehicle purchase (accounted for 88.1% of total cost per result) | Cost of mobile testing could be reduced by increasing test volumes, reducing input costs, or widening the test repertoire | Economic analysis with quantitative data; found mobile testing is more expensive than traditional lab testing; however, mobile testing holds potential for rapid case detection and improved coverage in high-burden communities |
| Cohen 2014 | South Africa | People with presumed TB, N = 403 participants | Urban hospital and clinic | Xpert | Centralized placement of Xpert led to delays for results due to laboratory processing time and time to transfer results from laboratory to clinic | As HIV care continues to decentralize in Africa, need to decentralize TB diagnostic capacity |  |
| Cordeiro-Santos 2020 | Brazil | People with productive cough of any duration; 2-year prospective study, N = 39 | Riverside communities on the outskirts of Manaus, Amazon rain forest | Xpert Ultra; Xpert Edge | Difficult-to-reach populations, such as riverside communities | Mobile boat | Brief report to assess feasibility |
| Das 2020 | India | Retrospective study of people with presumptive TB, N = 1042 | Conflict-affected tribal area | Xpert | Difficult-to-reach populations, such as people in conflict areas | Mobile testing strategy using Xpert with community health workers | MSF study; intersectoral measures such as access to a public distribution system, nutritional support, social welfare schemes, and security measures at the central and state levels will help to minimize the TB burden |
| Da Silva 2021 | Brazil | Presumed DR-TB with **≥** 1 of the following: a) failure of previous treatment, b) contact of DR-TB patient, c) PLHIV, d) homeless and/or hospitalized | Unified Health System Brazil | Xpert | Exchange rate depreciation may lead to limitations for incorporating technology into national health system; market factors, such as relations and/or changes in position of a developing country can lead to main cost component (cartridge) of the test assuming market value, ceasing to be cost-effective | Xpert is cost-effective with subsidies | Economic analysis; clinical trials advocated to assess new diagnostic tests, due to the potential to demonstrate organizational problems in local health systems |
| Da Silva Antunes 2014 | Brazil | Adult patients aged > 18 years with a diagnosis of pulmonary TB | Primary care clinics,  N = 6 | Xpert | Non-medical direct costs, such as transportation and food, and indirect costs, such as time spent for diagnostic visits | Transportation tickets, de-centralization of health care and cash transfer have been suggested | Economic analysis |
| Davids 2015 | South Africa | Survey, N = 400 health providers (doctors n = 255 and nurses n= 145) seeing patients with presumed TB | Primary care clinics | Xpert (and point-of-care tests in general) | Centralized laboratory services, poor QA, and lack of staff capacity; specific to TB: long laboratory TAT, difficulty in obtaining sputum samples, and lost results; complexity of healthcare system | Consider motivation and training of staff; evaluate way tests are put to use in daily practice | Survey was conducted during September 2012 and June 2013 |
| De Camargo 2015 | Brazil | Semi-structured interviews including people with TB (n = 30), health providers, laboratory technicians, managers | Urban clinic | Xpert | Concomitant implementation of new IT technology for recording and reporting test results | Decreased workload, and time and reliability of diagnosis |  |
| Engel 2015 | South Africa | 101 semi-structured interviews, and 7 focus group discussions with doctors, nurses, community health workers, patients, laboratory technicians, policymakers, hospital managers, and diagnostic manufacturers | Clinics and hospitals, rural and urban | Xpert (and other POC technologies) | Transporting samples and results in-between clinics/ hospitals and laboratories | Sufficient manpower and equipment, test result-based management decisions | Interviews not focused on TB diagnostics exclusively; importance of conducting operational research into health system requirements |
| England 2019 | Multi-country | Survey N = 16 | High burden countries with presence of MSF | Xpert | Cost; poor sensitization of clinical staff; high turnover of trained laboratory staff | Full implementation of WHO policy as a first step; sufficient service and maintenance provision provided by Cepheid to support optimization of existing instruments; and sustainable funding for the maintenance and expansion of networks |  |
| Gray 2016 | India, Peru, Uganda | Adults with symptoms suggestive of TB | Microscopy laboratories in urban and rural settings | TB-LAMP | Intensive training is required; proficiency training needed to be repeated at some sites; infrastructure concerns - two separate work areas are required to reduce the risk of DNA contamination; power supply; temperature control | Can reach higher throughput through the use of batched runs |  |
| Haraka 2015 | Tanzania | Retrospective cohort study of PLHIV with no reported TB history, N = 5123 | Rural clinic located in a hospital | Xpert | Vertical models of care; training and retention of staff; paper clinic logs | Integration of TB and HIV services together with a comprehensive electronic data collection with diagnosis using Xpert; systematic application of diagnostic algorithms |  |
| Joshi 2018 | Nepal | Focus group discussions, in-depth interviews, semi-structured interviews (patients), 23,075 Xpert tests in 21 diagnostic centres | Centres in district hospitals, primary health centres, district public health office laboratory | Xpert | Poor laboratory infrastructure;  need of incentive for staff; delays in cartridge supply, calibration and replacement of module; long travelling distance for patients | Training of trainers for maintenance and calibration of machine so that peripheral staff have capability; conducting community awareness and awareness with health staff so that eligible patients should be referred for Xpert test; expansion of machines | Implementation research using mixed methods sequential explanatory design; suggestion to conduct operational research before scale up |
| Kabugo 2021 | Uganda | Laboratory-based study | After pilot study, all TB laboratories in Uganda were included | Xpert | Technical incompetence to prepare panels, lack of infrastructure, lack of human resources, high cost to procure proficiency testing preparation equipment, lack of suitable means of transporting panels to laboratories, supply chain management breakages, poor connectivity and Information Technology | Quality management system, SOP for proficiency testing plan, on-site support, root cause analysis | The majority of the problems listed under root cause analysis were instantly fixed; fixing of actions that required many funds was never achieved in short times and affected the performance and routine effectiveness of the laboratories |
| Le 2019 | Vietnam | Retrospective analysis of hospital inpatient notes, ‘Xpert for all’ (DR-TB, PLHIV, adults and children with presumptive DS-TB) | Hospital inpatient setting, | Xpert | Resource limitations, sequence of Xpert testing | Integrate FAST into routine hospital activities to ensure sustainability with minimal additional work for already overburdened hospital staff; incorporate FAST process measures, such as time to initiation of likely effective anti-TB treatment | FAST strategy promotes symptom-based TB screening of hospitalised patients, followed by mWRD to inform prompt initiation of likely effective anti-TB treatment |
| McDowell 2016 | India | Private providers, N = 110; diversity of providers, including chest and bachelor of medicine and bachelor of surgery physicians, as well as physicians with training in Ayurveda, Unani, and homeopathy | Clinics, urban | TB tests including Xpert | Common practice of using empirical treatment; desire to provide rapid symptom relief; costs; uncertainty about the accuracy of available TB tests | Provider training and messaging; attention to providers’ care practices | Ethnographic study with interviews and observations. |
| Mnyambwa 2018 | Tanzania | Retrospective review of routine data of people with MDR-TB (verified by Xpert); key informant interviews: patients (N = 11), TB coordinators (N = 27) | Regional and district levels | Xpert | Some patients fear disclosure of their health condition to relatives; inaccurate patient data; incomplete information in the GxAlert database; superstition and stigma | GxAlert; harmonization of the recording system; training to all DOT nurses, TB coordinators, and laboratory technicians on the new diagnostic algorithm |  |
| Mustapha 2016 | Nigeria | Mixed methods study, Xpert laboratories | Secondary (N =10), tertiary (N = 10), private health facilities (N = 2) | Xpert | Absence of standardized sample transfer mechanism between sites with and without machines; non-adherence to protocols; frequent power interruption; long TAT; lack of awareness about role of Xpert | Decentralize services to primary health centers; training and supervision; consider solar as alternative power source; coordinate/integrate TB and HIV services; implement GxAlert; provider education | Data collected between September 2011 and December 2013 |
| Mwaura 2020 | Kenya and Swaziland | Focus group discussions with multiple stakeholders, focus group N= 47 participants and non-participant observation | Stakeholder meetings facilitated by FIND | Xpert MTB/RIF and Xpert Ultra | Through word of mouth, an individual’s false diagnosis could diminish the trust within his/her network in future diagnoses leading to unintended effects on adherence to medication for various diseases or increase attrition of presumptive TB cases; mismatch between number of cartridges used at the various Xpert sites and number of cartridges dispatched from national program | General attitude was that over-treating is better than under-diagnosing; improve reporting at local testing centres and use of the electronic information management system; de-centralize cartridge supply system | Considered trade-off between sensitivity and specificity of Xpert MTB/RIF and Ultra |
| Newtonraj 2019 | India | Interviews with 10 healthcare workers involved in implementation; medical officers/doctors n = 5, microbiologists n = 3, lab techs n = 2) | Xpert was located at the intermediate reference laboratory, district microscopy centres (mostly within district hospitals and medical colleges) would send samples of people eligible for testing | Xpert | Barriers included poor awareness among medical colleges and the private sector, difficulty in motivating sputum microscopy–negative patients for Xpert, and incompletely filled referral forms | Perceived benefits of Xpert were efficiency, rapid results, and detecting resistance. |  |
| Ntinginya 2021 | Kenya, Tanzania, and Uganda | Survey, participants N = 190 health officers | Districts and counties | Xpert | Insufficient funds; lack of awareness; procurement, inadequacy of utilities and human resources | Proposed national health tax and decentralizing management; proposed hub system with an effective sample referral network |  |
| Oliwa 2020 | Kenya | Interviews, small group discussions, and observations of child TB training, sensitization meetings, policy meetings, and hospital practices, and document review | County hospitals | Xpert | Sensitization was initially done for lab staff only; fear to make a diagnosis of TB in children sometimes due to stigma and TB-HIV association; difficulty of obtaining specimens in children | Behavior change interventions: training, modelling, persuasion, environmental restructuring, and education | The process resulted in a multi-faceted intervention package for redesigning child TB training; selection of champions; use of audit and feedback linked to group problem solving; and workflow restructuring with role specification |
| Oo 2019 | Myanmar | Mixed-methods study, analysis of routinely collected programme data and key informant interviews with health providers, N = 32 | Townships and 4 GeneXpert sites | Xpert | Lack of awareness of guidelines; human resource constraints and high work load; difficulties in specimen collection and transport to testing sites; travel difficulties; patients do not understand the importance of Xpert | Dissemination of the guidelines; dedicated human resource support; provide incentives or travel allowance to patients; communicate the importance of Xpert testing to patients |  |
| Pho 2015 | India | Model-based comparison of placement strategies for Xpert devices | 1,089 sites in the Uganda healthcare system that provided smear microscopy services for the diagnosis of TB from January 1 to December 31, 2011 | Xpert | Cost; lower case detection | Placement of the Xpert device in sites prioritized by high TB prevalence was superior to other strategies based on smear volume and EQA performance | Not an implementation study, analytical framework demonstrates value of combining operational decisions regarding site selection with clinical decisions regarding diagnostic algorithm and value of using program level data (e.g. smear volume, EQA performance) to inform critical decisions |
| Puri 2016 | Multi-country | TB experts in 12 countries were asked to check commercial availability of Xpert in their country and collect price data from private laboratories that offer Xpert testing, 2 or more respondents from each country | Country-wide | Xpert | The private sector is a major source of health care in 12 of the 22 countries with the highest TB burden; exclusion from concessional pricing programmes | Strategy needs to draw on various approaches: include private sector in current and future pricing agreements; replicate IPAQT-like models in other economies; consolidate private laboratories by public-private projects to so privately managed patients can be tested in public facilities; use subsidies and vouchers to cross-subsidize tests against more profitable tests | IPAQT - Initiative for Promoting Affordable and Quality TB Tests |
| Raizada 2014 | India | Feasibility study to assess ability of Xpert to return a valid patient result. | 18 Xpert study sites were selected to encompass range of diverse geographic and demographic settings across the country | Xpert | Not applicable | Not applicable; staff required minimal training; installation required only minor infrastructure modifications and provision of power backup; replacement of dysfunctional modules was possible (warranty or annual maintenance contract with the manufacturer); can use existing human resources and infrastructure found in public sector microscopy centres | Adequacy of public sector human resources, facilities, and temperature extremes proved unfounded; |
| Rendell 2017 | Mongolia | Semi-structured with laboratory staff, TB physicians, N = 24 | Urban, regional towns | Xpert | Poor awareness of guidelines; inadequate staffing; poor supply chain management of cartridges (stock-outs); lack of repair options; inconsistent training; paper-based system; poor sample quality | Clear guidelines in local language; extra staff; capacity for trouble shooting internally; access to experts and peer learning; knowledge of diagnostic algorithms; testing availability in provincial centres (decentralized) |  |
| Saria 2020 | India | Ethnographic study, with interviews and shadowing of formal and informal providers | Urban | Xpert | Providers very rarely tested for patients with TB on their first visit and instead offered antibiotics and other medicines; providers and technicians thought the test had high possibility of providing inaccurate results; challenging relations among the state, the market, and private providers; cost; providers were against Xpert replacing their expertise | When introducing a new technology, need to understand professional and social context at the local level | Not an implementation strategy but study provides rich data regarding provider and systems related issues of Xpert implementation in India |
| Shewade 2018 | India | Key informant interviews (N = 10) and focus group discussions (N = 2) with people involved in programmatic management of DR-TB | Urban, Xpert testing at tertiary district level facility; sputum smear at microscopy centre where samples need to be sent from (public) | Xpert | Delays in identification of patients eligible for testing due to use of treatment register as source for identifying patients; lack of assured specimen transport and tracking | Use of unique identifier at identification and throughout for tracking; improved specimen transport system; prompt feedback to microscopy centre |  |
| Sikhondze 2015 | Swaziland | A standardized laboratory monitoring and supervision checklist was used to assess the operational and functional aspects of Xpert in the TB diagnostic laboratories | Country-wide | Xpert | No dedicated government budget to support maintenance, annual calibration, modular replacements, and purchasing of warranty package | Ensure standardized methods of incorporating results into records; machines need to be functional all the time; therefore incorporate machine maintenance, etc. in country budgets; data need to be backed up centrally; remote monitoring systems need to be integrated into the routine Xpert implementation |  |
| Stevens 2017 | South Africa | Comment | Country-wide | Xpert | Programmatic: forecasting difficult;  equity issues;  clinical training lagged behind  laboratory implementation;  traditional and molecular algorithms used at the same time; nonadherence to diagnostic  algorithm  Quality: massive scale-up of quality assurance material  required  Laboratory: stock shortages, module failures, need for real-time monitoring, testing for extrapulmonary TB, testing children | Programmatic: Global funding, training, investments; Quality: development of verification program; Laboratory: focused on lab capacity, stock and workflow management, remote connectivity system, SOPs for extrapulmonary TB, added specimens for children | Not an implementation study |
| Tovar 2022 | Peru | Report describing initial 2 weeks of integrated screening for TB and COVID via mobile van, N = 672 | Urban | Ultra | People wanted test for COVID rather than test for TB; when on-site SARS-CoV-2 testing stopped, number of people decreased; acceptance of TB screening was a challenge, as people argued that their prolonged cough was due to COVID-19 rather than being at risk of TB | Communication and education strategies highlighting ability of radiography to detect both TB-related abnormalities and COVID-19 lesions led to increased acceptance of screening for both diseases | Optimization of screening |

Abbreviations: EQA: external quality assessment; FAST: Find cases Actively, Separate safely and Treat effectively; DOT: directly observed treatment; IPAQT: Initiative for Promoting Affordable and Quality TB Tests; POC: point of care; QA: quality assurance; SOP: standard operating procedures; Xpert: Xpert MTB/RIF
