# Supplementary material for: Implementation strategies to increase the uptake and impact of molecular WHO-recommended rapid diagnostic tests: evidence from a mixed-methods systematic review
Source: BMJ Glob Health. 2025 Sep 17;10(9):e018700. doi: 10.1136/bmjgh-2024-018700 (PMC12458786; doi:10.1136/bmjgh-2024-018700)
Supplement: online supplemental file 6 [file bmjgh-10-9-s006.docx]

**Table S4. Characteristics of studies of mWRD implementation, thin studies**

| **Study** | **Design** | **Country** | **mWRD** | **Insights** | **Comments** |
| --- | --- | --- | --- | --- | --- |
| Agizew 2019 | Systematic review | Multi-country, mainly Africa | Xpert | Consider interventions at 3 stages:  1. Pre-diagnostic  - training of Xpert operators, proficiency tests, assessment of potential diagnostic delays (patient health care seeking) and health system (sample transport), assess optimal placement of GeneXpert (high-burden areas, peripheral clinics).  2. Diagnostic  - maintenance, use latest version of Xpert cartridges (currently G4 or Ultra)  3. Post-diagnostic  - decrease TAT, enable early treatment initiation, reduce empiric treatment, track patients to reduce loss to follow-up |  |
| Alvarez 2015 | Prospective feasibility study | Canada | Xpert | Onsite placement of Xpert was feasible and could play an important role in improving TB diagnosis and health equity in a predominantly Inuit population in a remote, high-burden area | Onsite placement of Xpert feasible for reaching a vulnerable population |
| Antunes 2014 | Economic analysis | Brazil | Xpert | TB diagnosis and treatment has been decentralized to district clinics, but patient out-of-pocket expenses can still be a barrier to appropriate care. Transportation costs are important drivers of costs in peri-urban areas | Transportation in peri-urban areas add to out of pocket costs for patients |
| Cazabon 2017 | Survey questionnaire comparison between 2104 and 2015, correspondence | 22 high TB burden countries | Xpert | Though most countries had expanded diagnostic algorithms for Xpert, much more can be done to reach scale |  |
| Clouse 2017 | Observational cohort study and cross-sectional study to evaluate field implementation of Xpert | 30 participating International epidemiologic Databases to Evaluate AIDS (IeDEA) sites in 18 countries | Xpert | Researchers hypothesized that low utilization was due to cartridge stock-outs, interruptions in electricity, and unreliable transportation of specimens at sites with off-site Xpert testing; local study site investigators reported that provider education on WHO guidelines and importance of Xpert utilization may be lacking | Unreliable specimen transport |
| Codlin 2018 | Project data were analyzed providing descriptive statistics about people tested with Xpert and those diagnosed with TB | Cambodia | Xpert | This intervention did more than implement a new diagnostic tool. Community engagement activities increased the number of people evaluated for TB, in particular, elderly people in rural communities | Importance of community engagement for hard-to-reach populations |
| Creswell 2015 | Comparison of Xpert at NTP facilities versus referral to testing site | Nepal | Xpert | Improving the accuracy of diagnostic tests in a passive system may not be sufficient to increase TB case notification owing to high rates of empirical treatment | Need to improve access and access to services and test more people for TB |
| Doulla 2020 | Prospective pilot study of the revised surveillance system for previously treated TB cases | Tanzania | Xpert | Found shorter transit times and turnaround times; use of social media led to close follow-ups and timely response to concerns during the pilot | Quality improvement intervention |
| Du 2020 | Aim of this study was to develop a novel external quality assessment panel for molecular TB diagnostics | China | Xpert and TB-LAMP | Results suggest that molecular diagnostics may provide more reliable results than culture in resource-limited settings | Quality assurance |
| Fenner 2013 | On-line questionnaire survey of ART programs treating PLHIV | Sub-Saharan Africa, Asia, Latin America | Xpert | Some diagnostics, in particular Xpert when available, were more frequently used when offered for free. Even if price of test was reduced, patient costs may still be prohibitive in relation to their budget. Having free TB diagnostics appears to be important for the successful implementation of TB control in the PLHIV | Patients face difficult choices when balancing the costs of diagnostics with other demands on the family budget |
| Gupta 2014 | Retrospective analysis (audit) of cases where Xpert was used | UK | Xpert | High cost prohibited use of Xpert resulting in limited implementation in high resource, low incidence setting at this time |  |
| Hoang 2015 | Mixed method study that assessed reports and guidance, supplemented by focus group discussions and interviews | Vietnam | Xpert | Delayed funding, stock-outs, and difficulties with mail service affected Xpert implementation |  |
| Mohammed 2020 | Cross-sectional study supplemented by semi-structured interviews with selected health care workers and heads of health facilities | Ethiopia | Xpert | Knowledge gap, interruption of supplies (cartridges and reagents) affected implementation |  |
| Moyenga 2015 | Report on experience of introducing Xpert though a centralized approach | Burkina Faso | Xpert | HIV and TB programmes need to intensify their collaboration and link with the laboratory. In addition, appropriate coordination is necessary between the laboratory and pediatric providers and the laboratory and the penal system |  |
| Naidoo 2015 | Comparison of Xpert at NTP facilities versus referral to testing site; participants were part of a PROVE-IT observational cohort in 10 high TB-burden primary health-care facilities | South Africa | Xpert (ad other tests) | Barriers include: prception of poor public sector services which may have contributed both to deferred health-seeking and to patient’s use of the private sector, contributing to delays Enablers include: symptom recognition based on history of TB; social contact with TB/MDR-TB patients; awareness of increased risk of TB among PLHIV; convenience of free, accessible local services; family support | Each testing facility was provided with a generator, voltage stabilizer and an uninterrupted power supply device; solar panels were installed towards the end of the study period |
| Nakiyingi 2018 | Cross-sectional study, primarily of diagnostic accuracy | Uganda | TB-LAMP | Short training period required to run TB-LAMP; long power outages were managed with an uninterrupted power supply connected to the TB-LAMP machine | TB-LAMP can easily be performed and does not require sophisticated infrastructure and expertise |
| Oliwa 2018 | Study to assess determinants of use of Xpert | Kenya | Xpert | Xpert sensitization was initially done only for laboratory staff which  could have led to less demand for Xpert testing from clinicians; need increased attention on service delivery models that involve staff; increased staff education; address potential challenges of specimen referral with consideration for the private sector; improve record keeping | “Some studies identified that training, workload,  administrative support, staff motivation, role models and  participation in the guideline development influence the  implementation of TB guidelines in general.” |
| Piatek 2019 | Pre-COVID modelling of number of Xpert modules and funds needed to meet WHO goal of diagnosing and treating a cumulative 40 million people by 2022 | Global | Xpert | “These findings suggest that a major investment is needed in WRD capacity to implement the recommended diagnostic algorithm for TB.”  “Programmatic experience supports using a ratio of at least 10 adults tested with Xpert to 1 diagnosed TB patient.” | “However, it is clear from this analysis that countries do not have enough rapid TB test instruments or cartridges to meet their needs.” |
| Qin 2015 | Correspondence reporting on responses to questionnaire of national TB programme managers in 2013 | 22 high burden countries | Xpert | “We found current Xpert testing is mainly donor-funded, mostly limited to district or reference laboratories, and primarily used in patients suspected of having DR-TB.” Need greater investments from both donors and domestic funding. |  |
| Turaev 2021 | Analysis of aggregate data and secondary analysis of programme data from Tashkent City and Bukhara region | Uzbekistan | Xpert | Barriers: distance, especially for patients visiting primary care centers far away from the Xpert laboratories; difficulties with sample collection and transport systems; gaps in cartridge supply (lowing to lengthy custom clearances)  Enabler: possible presence of Médecins Sans Frontières, which provided technical and financial support |  |

Abbreviations: ART: antiretroviral therapy; NTP: National Tuberculosis Programme; Xpert: Xpert MTB/RIF
