# Supplementary material for: Implementation strategies to increase the uptake and impact of molecular WHO-recommended rapid diagnostic tests: evidence from a mixed-methods systematic review
Source: BMJ Glob Health. 2025 Sep 17;10(9):e018700. doi: 10.1136/bmjgh-2024-018700 (PMC12458786; doi:10.1136/bmjgh-2024-018700)
Supplement: online supplemental file 7 [file bmjgh-10-9-s007.docx]

**Table S5. Characteristics of operational reports with data on mWRD implementation strategies**

| **Agency** | **Title** | **Country** | **mWRD** | **Insights** | **Comments** |
| --- | --- | --- | --- | --- | --- |
| Coalition of Women Living with HIV and AIDS (COWLHA) and TAG (Treatment Action Group), 2022 | Community-Led Monitoring for Access to Tuberculosis Screening and Diagnosis Testing | Malawi | Xpert | -Community-led monitoring of tuberculosis programs are an important component in mWRD implementation  -Report increases the availability, accessibility, acceptability, and quality of TB testing and diagnosis | -Integrating communities as mWRD stakeholders can aid in monitoring |
| Department of Health & Family Welfare, Ministry of Health & Family Welfare, and Government of India, 2022 | 2021-2022 Annual Report | India | CBNAAT  Truenat | -Bi-directional Covid-19 and TB screening, diagnostic, and treatment capacity upgrades  -CBNAAT/Truenat instruments available in each district | -Multi-disease integrated testing |
| The Global Fund, 2022 | Fight for What Counts: Investment Case, | India | CBNAAT | -Integrated Covid-19 screening programs and laboratory services to conduct rapid onsite co-testing | - Multi-disease integrated testing |
| KNCV Tuberculosis Foundation, 2021 | KNCV Tuberculosis Foundation Annual Report 2021 | Global, Vietnam, Nigeria | Xpert | Reports on the development of self-paced e-modular training on in country diagnostic guidance delivery and diagnostic network optimization  -Reports on the use of the simple one-step (SOS) stool method using Xpert for TB diagnosis in children  -Reports on developing procedures and remote training materials for SOS stool for Xpert  -Reports on foundation support for Xpert maintenance and expansion in Nigeria by including new tools and new cartridges for the Xpert platform in both public and private sectors | Remote and modulate training, Multiple Disease Integration, Multisectoral Integration |
| STOP TB Partnership, 2021 | TB Screening in Mountain and Island Villages in Viet Nam Using Artificial Intelligence and Ultra-Portable X-Ray | Vietnam | Xpert | -Reports on the implementation of the “Double X Strategy” which use AI interpret digital x-rays to identify people for Xpert testing  -Reports on the mobile use of x-ray and Xpert technology | Technology integration |
| USAID, 2021 | A Time for Urgent Action To End TB: Tuberculosis Report To Congress | Nigeria | Xpert | -Reports on the creation of mobile diagnostic units combining Xpert and X-ray with AI powered by solar inverters and backup generators to screen 28,959 people  -Mobile units were adapted to dually screen and test for TB and Covid-19 in two states | Technology bundles, Multi-disease integrated testing |
| USAID, 2020 | Joint Assessment Report of TB Diagnostic Network Assessment | Vietnam | CBNAAT including Truenat | -Gaps in TB case detection  -Coordination of rapid TB diagnostic testing within comprehensive algorithms, only 25% of population lives within 5km radius of existing mWRD sites  -Reliable specimen referral system, particularly outside of NTP sites | Need to increase mWRD access, gaps in policy and guideline implementation |
| USAID, 2020 | Joint Assessment Report of TB Diagnostic Network Assessment | Zimbabwe | CBNAAT including Truenat | -Initial fees for chest x-ray  -While Ultra recommended as initial test, this is not implemented in private sector; trace result interpretation is challenging  - only 28% of population lives within 5km radius of existing mWRD sites  -Need for supportive supervision and quality performance indicator measurement | Bundled interventions that address functionality of mWRD network and decentralize mWRD access |
| USAID, 2019 | Joint Assessment Report of TB Diagnostic Network Assessment | Uganda | CBNAAT including Truenat | -Need to expand access to Xpert Ultra and reduce key cascade delays  -Supportive supervision to improve quality of laboratory testing  -Strengthened data collection  -Develop end-to-end integrated interoperable connected systems and electronic data systems | Bundled interventions, need to address patient pathways beyond laboratory aspects of algorithms, gaps in policy and guideline implementation |
| USAID, 2017 | Joint Assessment Report of TB Diagnostic Network Assessment | India | CBNAAT including Truenat | -Need for state specific performance improvement plans  -Need to address human resource crisis  -Supportive supervision  -Deploy electronic data systems, address usability issues of Nikshay system including user-oriented design, ability to handle large-volume data and improve server capacity | Variability in diagnostic network across country, translate public-private mix policy throughout diagnostic network |
| National Tuberculosis and Leprosy Control Programme at the Federal Ministry of Health Nigeria, Department of Public Health,2017 | National Tuberculosis and Leprosy Control Programme Public-Private Mix Action Plan | Nigeria | Xpert | Reports on early integration of public and private sector Xpert services in Nigeria through sample transportation, supporting the purchase of consumables, and placing instruments in private tertiary hospitals | Multi-sectoral integration |
| Revised National Tuberculosis Control Program (Indian Ministry of Health and Family Services), 2017 | National Strategic Plan for Tuberculosis Elimination 2017-2025 | India | CBNAAT, LPA, Truenat, Xpert | Outlines expansion of mWRD tools within the health system’s tiers, notes the expansion of IPAQT for all WHO-approved mWRDs | Integration of mWRDs across health systems levels and sectors |
| TB REACH, 2015 | Improving Tuberculosis Case Detection: A compendium of TB reach case studies, lessons learned and a monitoring and evaluation framework | Nigeria  Tajikistan  Nigeria  Cambodia  Uganda  South Africa | Xpert  Xpert  Xpert  Xpert  Xpert  Xpert | -Report on “retrofitting” hospital labs by renovating space for mWRD to increase TB detection  -Report on using mobile phone base technology to verbally screen for TB and Xpert testing  -Report on facilitating sample transportation through motobike transporters.  -Report that using generators and uninterrupted power supply devices as well as locating Xpert machines in cargo containers increased case detection rates  Combined Xpert with chest x-ray and verbal symptom screening to eliminate smear microscopy  Used an automated SMS system connected to GeneXpert to send tests to a centralized server to monitor failed test rates and facilitate targeted maintenance and training  Engaged health workers to transport sputum samples collected at home to Xpert machines | -mWRD infrastructural enablers  -Integrating technologies  -Built mWRD systems  -Integrated technologies  -Community-based sample transport and patient-centered care |
| STOP TB Partnership, Not Dated | Unmaned Aerial Vehicles: A Tool for Tuberculosis Care | Nepal | Xpert | Reports on the use of drones to carry samples from remote health facilities to laboratory hubs for GeneXpert testing | Integrating new technologies to increase mWRD access |

NTP: National Tuberculosis Programme; Xpert: Xpert MTB/RIF, CBANNAT: cartridge-based nucleic acid amplification test
