# Supplementary material for: Implementation strategies to increase the uptake and impact of molecular WHO-recommended rapid diagnostic tests: evidence from a mixed-methods systematic review
Source: BMJ Glob Health. 2025 Sep 17;10(9):e018700. doi: 10.1136/bmjgh-2024-018700 (PMC12458786; doi:10.1136/bmjgh-2024-018700)
Supplement: online supplemental file 8 [file bmjgh-10-9-s008.docx]

**Table S6. Assessment of methodological limitations using an adapted version of the Standards for Reporting Implementation Studies (StaRI).**

| **Study** | **Are the study rationale and aims well stated?** | **Are the design and context appropriate for the aims of the study?** | **Is the implementation strategy well described?** | **Were steps taken to increase rigour in analysis of the data?** | **Are the findings supported by the data?** | **Are the implications policy or practice discussed and justified?** |
| --- | --- | --- | --- | --- | --- | --- |
| **Abdurrahman 2015** | Yes, fairly thorough | Yes, fairly thorough | Yes, fairly thorough | Yes, in part | Yes, in part | Yes, fairly thorough |
| **Agizew 2017** | Yes, fairly thorough | Yes, fairly thorough | Yes, fairly thorough | Yes, fairly thorough | Yes, fairly thorough | Yes, fairly thorough |
| **Alagna 2020** | Yes, fairly thorough | Yes, fairly thorough | Yes, fairly thorough | Yes, fairly thorough | Yes, fairly thorough | Yes, fairly thorough |
| **Albert 2020** | Yes, fairly thorough | Yes, fairly thorough | Yes, fairly thorough | Yes, fairly thorough | Yes, fairly thorough | Yes, fairly thorough |
| **Awan 2018** | Yes, fairly thorough | Yes, fairly thorough | Yes, fairly thorough | Yes, fairly thorough | Yes, fairly thorough | Yes, fairly thorough |
| **Babirye 2019** | Yes, fairly thorough | Yes, fairly thorough | Yes, fairly thorough | Yes, fairly thorough | Yes, fairly thorough | Yes, fairly thorough |
| **Banu 2020** | Yes, fairly thorough | Yes, fairly thorough | Yes, fairly thorough | Yes, fairly thorough | Yes, fairly thorough | Yes, fairly thorough |
| **Brown 2021** |  |  |  |  |  |  |
| **Cattamanchi 2020** | Yes, fairly thorough | Yes, fairly thorough | Yes, fairly thorough | Yes, fairly thorough | Yes, fairly thorough | Yes, fairly thorough |
| **Cattamanchi 2021** | Yes, fairly thorough | Yes, fairly thorough | Yes, fairly thorough | Yes, fairly thorough | Yes, fairly thorough | Yes, fairly thorough |
| **Clouse 2012** | Yes, in part | No, not at all/Not stated/Can’t tell | Yes, fairly thorough | No, not at all/Not stated/Can’t tell | Yes, in part | Yes, in part |
| **Colvin 2015** | Yes, fairly thorough | Yes, fairly thorough | Yes, fairly thorough | Yes, fairly thorough | Yes, fairly thorough | Yes, fairly thorough |
| **Cowan 2015** | Yes, in part | Yes, fairly thorough | Yes, fairly thorough | Yes, in part | Yes, fairly thorough | Yes, fairly thorough |
| **Cowan 2016** | Yes, fairly thorough | Yes, fairly thorough | Yes, fairly thorough | Yes, fairly thorough | Yes, fairly thorough | Yes, fairly thorough |
| **Creswell 2014** | Yes, fairly thorough | Yes, fairly thorough | Yes, fairly thorough | Yes, fairly thorough | Yes, fairly thorough | Yes, fairly thorough |
| **Dabas 2019** | Yes, fairly thorough | Yes, fairly thorough | Yes, fairly thorough | Yes, in part | Yes, fairly thorough | Yes, fairly thorough |
| **Deo 2020** | Yes, fairly thorough | Yes, fairly thorough | Yes, fairly thorough | Yes, fairly thorough | Yes, fairly thorough | Yes, fairly thorough |
| **Deo 2021** | Yes, fairly thorough | Yes, fairly thorough | Yes, fairly thorough | Yes, fairly thorough | Yes, fairly thorough | Yes, fairly thorough |
| **Durovni 2014** | Yes, fairly thorough | Yes, fairly thorough | Yes, fairly thorough | Yes, fairly thorough | Yes, fairly thorough | Yes, fairly thorough |
| **Durovni 2014** | Yes, fairly thorough | Yes, fairly thorough | Yes, in part | No, not at all/Not stated/Can’t tell | Yes, in part | Yes, fairly thorough |
| **Engel 2022** |  |  |  |  |  |  |
| **Gidado 2018** | Yes, fairly thorough | Yes, in part | Yes, fairly thorough | Yes, in part | Yes, fairly thorough | Yes, fairly thorough |
| **Hanrahan 2016** | Yes, fairly thorough | Yes, fairly thorough | Yes, fairly thorough | Yes, fairly thorough | Yes, fairly thorough | Yes, fairly thorough |
| **Jeyashree 2020** | Yes, fairly thorough | Yes, fairly thorough | Yes, fairly thorough | Yes, fairly thorough | Yes, fairly thorough | Yes, fairly thorough |
| **Khushvakhtov 2021** | Yes, fairly thorough | Yes, fairly thorough | Yes, fairly thorough | Yes, fairly thorough | Yes, fairly thorough | Yes, fairly thorough |
| **Lessells 2017** | Yes, fairly thorough | Yes, fairly thorough | Yes, fairly thorough | Yes, fairly thorough | Yes, in part | Yes, fairly thorough |
| **Lisboa 2020** | Yes, fairly thorough | Yes, fairly thorough | Yes, fairly thorough | Yes, fairly thorough | Yes, fairly thorough | Yes, fairly thorough |
| **Manabe 2015** | Yes, fairly thorough | Yes, fairly thorough | Yes, fairly thorough | Yes, fairly thorough | Yes, fairly thorough | Yes, fairly thorough |
| **McDowell 2018** | Yes, fairly thorough | Yes, fairly thorough | Yes, fairly thorough | Yes, fairly thorough | Yes, fairly thorough | Yes, fairly thorough |
| **Medina-Marino 2021** | Yes, fairly thorough | Yes, fairly thorough | Yes, fairly thorough | Yes, fairly thorough | Yes, fairly thorough | Yes, fairly thorough |
| **Nalugwa 2020** | Yes, fairly thorough | Yes, fairly thorough | Yes, fairly thorough | Yes, in part | Yes, fairly thorough | Yes, fairly thorough |
| **Nalugwa 2022** | Yes, fairly thorough | Yes, fairly thorough | Yes, fairly thorough | Yes, fairly thorough | Yes, fairly thorough | Yes, fairly thorough |
| **Nathavitharana 2017** | Yes, fairly thorough | Yes, in part | Yes, fairly thorough | Yes, in part | Yes, in part | Yes, fairly thorough |
| **Ndlovu 2018** | Yes, fairly thorough | Yes, fairly thorough | Yes, fairly thorough | Yes, in part | Yes, fairly thorough | Yes, fairly thorough |
| **Ngwira 2019** | Yes, fairly thorough | Yes, fairly thorough | Yes, fairly thorough | Yes, fairly thorough | Yes, fairly thorough | Yes, fairly thorough |
| **Page-Shipp 2014** | Yes, in part | Yes, in part | Yes, fairly thorough | No, not at all/Not stated/Can’t tell | Yes, in part | Yes, in part |
| **Paudel 2021** | Yes, fairly thorough | Yes, fairly thorough | Yes, fairly thorough | Yes, fairly thorough | Yes, fairly thorough | Yes, fairly thorough |
| **Pho 2015** | Yes, fairly thorough | Yes, fairly thorough | Yes, fairly thorough | Yes, fairly thorough | Yes, fairly thorough | Yes, fairly thorough |
| **Raizada 2015** | Yes, fairly thorough | Yes, fairly thorough | Yes, fairly thorough | Yes, in part | Yes, fairly thorough | Yes, fairly thorough |
| **Raizada 2018** | Yes, fairly thorough | Yes, fairly thorough | Yes, fairly thorough | Yes, in part | Yes, fairly thorough | Yes, fairly thorough |
| **Raizada 2018** | Yes, in part | Yes, fairly thorough | Yes, fairly thorough | Yes, in part | Yes, fairly thorough | Yes, fairly thorough |
| **Raizada 2018** | Yes, fairly thorough | Yes, fairly thorough | Yes, fairly thorough | Yes, in part | Yes, fairly thorough | Yes, fairly thorough |
| **Raizada 2021** | Yes, fairly thorough | Yes, fairly thorough | Yes, fairly thorough | Yes, fairly thorough | Yes, fairly thorough | Yes, fairly thorough |
| **Reza 2020** | Yes, fairly thorough | Yes, fairly thorough | Yes, fairly thorough | Yes, fairly thorough | No, not at all/Not stated/Can’t tell | Yes, in part |
| **Schumacher 2015** | Yes, fairly thorough | Yes, fairly thorough | Yes, fairly thorough | Yes, fairly thorough | Yes, fairly thorough | Yes, fairly thorough |
| **Shete 2017** | Yes, fairly thorough | Yes, fairly thorough | Yes, fairly thorough | Yes, fairly thorough | Yes, fairly thorough | Yes, fairly thorough |
| **Shibu 2020** | Yes, fairly thorough | Yes, fairly thorough | Yes, fairly thorough | Yes, in part | Yes, in part | Yes, fairly thorough |
| **Stime 2018** | Yes, fairly thorough | Yes, fairly thorough | Yes, fairly thorough | Yes, fairly thorough | Yes, fairly thorough | Yes, fairly thorough |
| **Theron 2014** | Yes, fairly thorough | Yes, fairly thorough | Yes, fairly thorough | Yes, fairly thorough | Yes, fairly thorough | Yes, fairly thorough |
| **Umubyeyi 2016** | Yes, in part | Yes, in part | Yes, in part | No, not at all/Not stated/Can’t tell | Yes, in part | Yes, in part |
| **VandenHandel 2015** | Yes, fairly thorough | Yes, fairly thorough | Yes, fairly thorough | Yes, fairly thorough | Yes, fairly thorough | Yes, fairly thorough |
| **Vatsyayan 2022** | Yes, fairly thorough | Yes, fairly thorough | Yes, fairly thorough | No, not at all/Not stated/Can’t tell | Yes, in part | Yes, fairly thorough |
| **Yuen 2021** | Yes, fairly thorough | Yes, fairly thorough | Yes, fairly thorough | Yes, fairly thorough | Yes, fairly thorough | Yes, fairly thorough |
| **Zawedde-Muyanja 2022** | Yes, fairly thorough | Yes, fairly thorough | Yes, fairly thorough | Yes, fairly thorough | Yes, fairly thorough | Yes, fairly thorough |
| **Zishiri 2015** | Yes, fairly thorough | Yes, fairly thorough | Yes, fairly thorough | Yes, fairly thorough | Yes, fairly thorough | Yes, fairly thorough |
